# Comparative effectiveness and cost-effectiveness of *Chuna* manual therapy versus conventional usual care for nonacute low back pain: study protocol for a pilot multicenter, pragmatic randomized controlled trial (pCRN study)

**DOI:** 10.1186/s13063-016-1756-8

**Published:** 2017-01-17

**Authors:** Byung-Cheul Shin, Me-riong Kim, Jae-Heung Cho, Jae-Young Jung, Koh-Woon Kim, Jun-Hwan Lee, Kibong Nam, Min ho Lee, Eui-Hyoung Hwang, Kwang-Ho Heo, Namkwen Kim, In-Hyuk Ha

**Affiliations:** 1 Spine and Joint Center, Pusan National University Korean Medicine Hospital, Yangsan, Republic of Korea; 2 Department of Korean Rehabilitation Medicine, School of Korean Medicine, Pusan National University, Yangsan, Republic of Korea; 3 Jaseng Spine and Joint Research Institute, Jaseng Medical Foundation, 858 Eonju-ro, Gangnam-gu, Seoul, 135-896 Republic of Korea; 4 Department of Korean Rehabilitation Medicine, Kyung Hee University, Seoul, Republic of Korea; 5 Clinical Research Division, Korea Institute of Oriental Medicine, Daejeon, Republic of Korea; 6 Korean Medicine Life Science, University of Science and Technology (UST), Campus of Korea Institute of Oriental Medicine, Daejeon, Republic of Korea; 7 Mokhuri Neck and Back Hospital, Seoul, Republic of Korea; 8 Center for Comparative Effectiveness Research and Economic Evaluation in Korean Medicine, Pusan National University, Yangsan, Republic of Korea

**Keywords:** Low back pain, Spinal manipulation, Cost-benefit analysis, Complementary therapies, Protocol

## Abstract

**Background:**

While *Chuna* manual therapy is a Korean manual therapy widely used primarily for low back pain (LBP)-related disorders in Korea, well-designed studies on the comparative effectiveness of *Chuna* manual therapy are scarce.

**Methods/design:**

This study is the protocol for a three-armed, multicenter, pragmatic randomized controlled pilot trial. Sixty severe nonacute LBP patients (pain duration of at least 3 weeks, Numeric Rating Scale (NRS) ≥5) will be recruited at four Korean medicine hospitals. Participants will be randomly allocated to the *Chuna* group (*n* = 20), usual care group (*n* = 20), or *Chuna* plus usual care group (*n* = 20) for 6 weeks of treatment. Usual care will consist of orally administered conventional medicine, physical therapy, and back pain care education. The trial will be conducted with outcome assessor and statistician blinding. The primary endpoint will be NRS of LBP at week 7 post randomization. Secondary outcomes include NRS of leg pain, the Oswestry Disability Index (ODI), the Patient Global Impression of Change (PGIC), the Credibility and Expectancy Questionnaire, lumbar range of motion (ROM), the EuroQol-5 Dimension (EQ-5D) health survey, the Health Utility Index III (HUI-III), and economic evaluation and safety data. Post-treatment follow-ups will be conducted at 1, 4, and 10 weeks after conclusion of treatment.

**Discussion:**

This study will assess the comparative effectiveness of *Chuna* manual therapy compared to conventional usual care. Costs and effectiveness (utility) data will be analyzed for exploratory cost-effectiveness analysis. If this pilot study does not reach a definite conclusion due to its small sample size, these results will be used as preliminary results to calculate sample size for future large-scale clinical trials and contribute in the assessment of feasibility of a full-scale multicenter trial.

**Trial registration:**

Clinical Research Information Service (CRIS), KCT0001850. Registered on 17 March 2016.

**Electronic supplementary material:**

The online version of this article (doi:10.1186/s13063-016-1756-8) contains supplementary material, which is available to authorized users.

## Background

### Background and rationale

Low back pain (LBP) is a highly prevalent musculoskeletal disorder that incurs severe pain, increase in sick leave and substantial social costs [1], is disabling to both individual and society and affects approximately 70–80% adults over their lifetimes [2]. Therefore, appropriate LBP treatment selection is of considerable importance for individual patients, physicians, and decision-makers of health care policy.

Spinal manipulative therapy (SMT) is extensively used for acute and chronic LBP, and its efficacy has been reported in numerous randomized controlled trials (RCTs) and systematic reviews (SRs). However, upon closer inspection, the two Cochrane reviews on SMT for acute and chronic LBP mainly use search terms such as manipulation, osteopathy, and chiropractic, and do not include Korean *Chuna* manual therapy or Chinese *Tuina* [3]. Moreover, it can be reasonably inferred that though SMT is widely recommended for both acute and chronic LBP in various guidelines [4–6], the vast disparity in background history, diagnosis and treatment principles and techniques precludes unitary appreciation of treatment effect.

*Chuna*, literally meaning manual treatment in Korean, is a Korean spinal manipulation that has developed absorbing aspects of Chinese *Tuina*, chiropractic manipulation, and osteopathic medicine over time. Manipulative therapy can, therefore, be likened to a reservoir rich with academic contributions from various health care disciplines, drawing from, and emptying into, reserves to form a continuous cycle. *Chuna* incorporates radiology-based diagnosis, which is not customary for Chinese SMT, and includes meridian theory, meridian muscle concepts, and organ pattern identification diagnosis not usually seen in chiropractic manipulation or osteopathic medicine [7]. However, a recent study assessing the *Chuna* literature published from 1995 to May 2013 in Korea reported that clinical studies mainly consisted of case series (*n* = 99; 72%), RCTs (*n* = 15; 11%), and non-RCTs (*n* = 21; 16%) [8]. While the proportion of RCTs in *Chuna*-related studies is steadily increasing, most studies take on an integrative approach as opposed to single treatment, and are not well-equipped to rigorously evaluate its therapeutic effect. Also, in quality assessment of RCTs in accordance with the Consolidated Standards of Reporting Trials (CONSORT) Statement [9], weaknesses regarding identification in title or structured summary in abstract, inadequate trial design description, statistical limitation from small sample size, inaccuracy of randomization and allocation process, difficulty in blinding, and insufficient reporting of participant flow and estimated effect size of interventions and its precision were reported [10].

While *Chuna* manual therapy is primarily used for LBP in Korea with high patient satisfaction [11], it is currently not covered by national health insurance and thus accessibility due to cost is limited considering its high satisfaction rate [12]. The Korean Ministry of Health and Welfare’s 2011 report on usage of Korean medicine shows that *Chuna* was one of the most common usages of Korean medicine [11]. At the 2011 National Assembly forum for insurance guarantee reinforcement of Korean medicine, increasing national insurance coverage of Korean medicine treatments, such as *Chuna* manual therapy, was proposed with the aim of increasing accessibility to Korean medicine services, promoting national health, and improving quality of life [13]. Therefore, well-designed, high-quality RCTs are highly needed in the research sector to determine the effectiveness and economic value of *Chuna* manual therapy and to meet social demand.

### Objectives

Objective evaluation of the comparative effectiveness, safety, and economic evaluation of *Chuna* manual therapy for application in cost-effective, evidence-based practice are important issues both for the Korean medicine sector and for national health services improvement. High-quality RCTs are warranted for rigorous assessment of *Chuna* effectiveness, safety, and cost-effectiveness. The hypotheses of this study are as follows: (1) *Chuna* manual therapy will be more effective and safer than (or equally safe as) conventional usual care in nonacute LBP patients and (2) concurrent treatment consisting of *Chuna* manual therapy and usual care will be more effective than *Chuna* alone or conventional usual care alone in nonacute LBP patients. Therefore, the objectives of this pCRN (pilot *Chuna* Research Network) study are to investigate the following in nonacute LBP patients: (1) the comparative effectiveness and safety of *Chuna* manual therapy compared to conventional usual care through comparison of pain, functionality, quality of life parameters and adverse events, (2) the effectiveness and safety of a concurrent treatment consisting of *Chuna* manual therapy and usual care, which is reflective of actual clinical practice, through comparison of two single treatment groups of *Chuna* manual therapy and usual care, and a combined treatment group of *Chuna* manual therapy with usual care and (3) costs and effectiveness (utility) data of the three groups for exploratory cost-effectiveness analysis (economic evaluation). However, if the current sample size deters this pilot study from reaching a definite conclusion regarding aims (1), (2), and (3), the main objectives of this preliminary study will be to determine a sample size appropriate for statistical testing in future large-scale studies and to assess the feasibility of a full-scale multicenter trial.

## Methods/design

The protocol of this pCRN study is presented in accordance with the 2013 SPIRIT (Standard Protocol Items: Recommendations for Interventional Trials) Statement (see Additional file 1 for the populated SPIRIT Checklist and Table 1 for the trial schedule of enrollment, interventions, and assessments in accordance with recommended SPIRIT figure).

**Table 1:** Time points of each assessment index

	Study period										
Time point	Enrollment	Allocation	Active treatment post allocation						Follow-up		
	Week −1	Week 0 (Baseline)	Week 1	Week 2	Week 3	Week 4	Week 5	Week 6	Week 7	Week 10	Week 14
Enrollment											
Eligibility screening	○										
Written informed consent	○										
Vital signs	○								○	○	○
Sociodemographic characteristics, medical history (e.g., LBP, medication history)	○										
Physical examination	○										
Randomized allocation		○									
Interventions											
Treatment in CMT group			←2–3 times/week →				←1–3 times/week →				
Treatment in UC^a^ group (times/week)			←2–3 times/week →				←1–3 times/week →				
Treatment in CMT + UC^a^ group (times/week)			←2–3 times/week →				←1–3 times/week →				
Assessments											
Symptoms and change in medicine		○		○	○	○	○	○	○	○	
NRS of LBP and leg pain	○	○		○	○	○	○	○	○	○	○
ODI		○					○		○	○	
PGIC							○		○	○	
ROM		○					○		○	○	
EQ-5D		○					○		○	○	○
HUI-III		○					○		○	○	○
Economic evaluation		○					○		○	○	○
Adverse events		○	← every visit →						○	○	○

*CMT Chuna* manual therapy, *UC* usual care, *NRS* Numeric Rating Scale, *LBP* low back pain, *ODI* Oswestry Disability Index, *PGIC* Patient Global Impression of Change, *ROM* range of movement, *EQ-5D* EuroQol-5 dimensions, *HUI-III* Health Utility Index III

^a^UC: medication will be administered daily and physical therapy will be applied 2–3 times/week

### Trial design and study setting

This pCRN study employs a multicenter, randomized, three-armed, parallel-group design. Participants will be randomly allocated to the *Chuna* manual therapy group, a usual care group, or a combined treatment group of *Chuna* manual therapy with usual care at a ratio of 1:1:1. Participants will be recruited at four major Korean medicine hospitals in Korea (Pusan National University Korean Medicine Hospital, Kyung Hee University Korean Medicine Hospital at Gangdong, Jaseng Hospital of Korean Medicine, and Mokhuri Neck and Back Hospital) and recruitment period is anticipated to be from February to August 2016. Participants will be recruited through each hospital website and online and offline advertisements. These study sites possess the facilities, personnel, and patient recognition required for LBP patient recruitment, and further information on the study sites is available at Clinical Research Information Service (CRIS registration number: KCT0001850). Participants will receive a total 6 weeks of outpatient treatment for LBP starting from visit 2 through random allocation if deemed eligible in screening at visit 1. General management and treatment processes will adhere to predetermined clinical trial procedures.

The entire research process, design, and setting were prepared through six or more meetings of 22 pCRN members including rehabilitation experts (*n* = 14), clinical research coordinators (*n* = 4), clinical research methodologists (*n* = 2), and economic evaluation experts (*n* = 2) currently employed at six university hospitals, two spine-specialty Korean medicine hospitals, and the Korea Institute of Oriental Medicine (see Additional file 2 for trial committee organization and contributions and role).

### Participants

#### Inclusion criteria

1. Nonacute LBP participants (with pain duration of 3 weeks or longer) requiring medical attention
2. Participants with average Numeric Rating Scale (NRS) ≥5 during the past week
3. Participants aged 19 years or older, and 70 years or younger
4. Participants who have agreed to trial participation and provided written informed consent

#### Exclusion criteria

1. Participants diagnosed with serious pathology(s) which may cause LBP (e.g., spinal metastasis of tumor(s), acute fracture, spinal dislocation)
2. Participants with spinal surgery history within the last 3 months
3. Participants with other chronic disease(s) which may interfere with treatment effect or outcome interpretation (e.g., chronic renal failure)
4. Participants with progressive neurologic deficit or severe neurologic symptoms
5. Patients with an inner fixation or stabilization device mounted through spinal surgery
6. Participants currently taking steroids, immunosuppressants, medicine for psychological problems or other medication(s) that may interfere with study results
7. Participants who have received *Chuna* manual therapy or medicine which may influence pain levels, such as nonsteroidal anti-inflammatory drugs (NSAIDs), within the past week
8. Pregnant participants or participants who are planning to become pregnant
9. Subjects participating in other clinical studies or who are otherwise deemed unsuitable by the researchers

### Randomization and allocation concealment

A statistician blinded to the objectives and exact design of this study will use SAS 9.3 to generate the allocation sequence using block randomization (block size = 6). Researchers will take care so that block sizes do not become known to the researchers who conduct participant recruitment or intervention allocation.

The researcher conducting participant recruitment at each site (Korean medicine doctor (KMD)) will contact the main trial site for central randomization by phone for random allocation to the three groups at a ratio of 1:1:1 of participants who have voluntarily agreed to participate in the trial, given written informed consent, and been screened and found eligible for trial participation in accordance with inclusion and exclusion criteria. Central randomization by phone will be performed through contact upon participant enrollment by the researcher conducting participant recruitment at each site to a designated independent researcher at the main trial site who will consecutively assign the randomized number and allocation group of each participant by verifying the sequence in the order of phone contact. All allocation numbers will be concealed through blinding of randomized number generation and central phone randomization.

The four sites will receive random allocation competitively on participant enrollment without stratification by site, and each site will respectively stop recruitment when the equal allocated number of 15 participants has been recruited.

### Blinding

As blinding of physicians and participants to allocation of treatment groups is impossible in the current design due to dissimilarity of interventions, only outcome assessors, statistician and data analysts will be blinded. The outcome assessors will not participate in treatment and a research nurse or KMD blinded to allocation will conduct outcome assessment in a separate room after treatment and will not attempt to become knowledgeable of allocation. The patient will also be cautioned against informing the outcome assessor of treatment allocation prior to each assessment. The electronic data transferred to statistician and data analysts will not contain information on treatment allocation.

### Interventions

#### Chuna manual therapy (see Additional file ***3*** for technical details with photos)

A semistandardized *Chuna* treatment plan for LBP was established by technique selection based on specialist opinion collected through survey of *Chuna* clinical specialists (total *n* = 20) [14] and physicians employed at a spine-specialty designated Korean medicine hospital [15]. The physicians administering *Chuna* in this trial will be KMDs with 3+ years’ clinical experience of *Chuna* manual therapy use, and will be deployed after receiving *Chuna* protocol training sessions (two sessions, 4+ h/session) for standardized application. *Chuna* covers a wide variety of techniques including high-velocity, low-amplitude thrusts to joints, slightly further than the passive range of motion, and mobilization which applies manual force to joints in the passive range of motion [16]. The *Chuna* techniques utilized in this study are divided into lumbar and pelvic regions, and mandatory or selective techniques will be performed following physician judgement (Table 2).

**Table 2:** Semistandardized treatment plan of study *Chuna* techniques [38–41]

Region	Mandatory techniques	Selective techniques
Lumbar spine^a^	• Spine flexion distraction technique (flexion) • Sidelying lumbar (extension or flexion) dysfunction correction technique	• Spinal flexion distraction (circumduction) • Spinal flexion distraction (sidebending) • Iliopsoas fascial *Chuna*
Pelvis^a^	<Iliac dysfunction (if applicable by evaluation) > • Prone iliac anterior rotation dysfunction correction technique/ or • Prone iliac posterior rotation/sacral sidebending dysfunction correction technique • Prone leg raise iliac dysfunction correction technique • Prone inflare-outflare dysfunction correction technique < Sacral dysfunction (if applicable by evaluation) > • Prone sacral sidebending and rotation dysfunction correction technique	• Prone sacral dysfunction correction technique (extension or flexion)

^a^Dysfunction type assessed and diagnosed prior to treatment

A total of 10–18 *Chuna* manual therapy sessions (at least 10 sessions or more are required to be received by participants) will be administered over a 6-week period. Frequency of treatment will differ by period with two to three sessions/week from week 1 to week 4, and one to three sessions/week from week 5 to week 6 based on *Chuna* physician judgement of previous treatment results. The time duration of one *Chuna* manual therapy session will be approximately 15 min including evaluation and treatment.

#### Usual care

Conventional usual care will be limited to orally administered medicine, physical therapy, and back pain care education (15-min education with visual aids covering cause, prevention, management, and exercise for back pain care) in this study. Medication and physical therapy of usual care will be provided with reference to most common treatments used in LBP-related patients as assessed from 2011 Korean Health Insurance Review and Assessment (HIRA) statistics [17] or clinical practice guidelines, provided as lists of most frequent medicine and physical treatments to the usual care physician [5]. Participants will be asked to record drug intake to monitor adherence, and medicine and physical therapy usage type and frequency will be recorded in a separate Case Report Form (CRF) for outcome assessor blinding purposes.

#### Combined Chuna manual therapy and usual care

The combined treatment group of *Chuna* manual therapy and usual care will receive both treatments in the same method, frequency, session length, total duration, and number of sessions of each treatment as that of the other two groups.

#### Cointerventions

Additional treatment (e.g., other medication related to pain, acupuncture, procedures or surgery) other than that specified in the protocol will not be allowed during the 6-week intervention period.

### Outcomes

#### Primary outcome measurement

The primary outcome will be LBP level for the past week assessed using the NRS. In the NRS, the participant selects a number from 0 to 10 that best indicates current pain level (with 0 indicating no pain, and 10 the most severe pain one can imagine) [18, 19].

#### Secondary outcome measures

Secondary outcome measures include leg pain level for the past week as assessed using the NRS. Functional status will be evaluated using the Oswestry Disability Index (ODI) questionnaire. The ODI is a 10-item questionnaire developed for the purpose of LBP-related disability assessment [20]. Each item has a six-level answer choice, and each level correlates with a score of 0–5. Higher scores reflect greater disability. The accredited Korean version of the ODI [21] will be used. The Patient Global Impression of Change (PGIC) will be assessed for comprehensive and global assessment of change in LBP and movement limitation due to pain [18, 22]. The PGIC discerns subjective impression of change in seven levels ranging from 1 = very much improved; 2 = much improved; 3 = minimally improved; 4 = no change; 5 = minimally worse; 6 = much worse; to 7 = very much worse. While the PGIC was initially developed for psychological use, it is currently applied in pain evaluation of various medical fields. The EuroQol-5 dimensions (EQ-5D) health survey is a measurement tool for health-related quality of life and widely used across the health care sector. The range of scores is from −1, indicating health considered worse than death to 1, meaning perfect health [23]. The EQ-5D comprises five dimensions or items assessing current health state, which are mobility, self-care, usual activities, pain/discomfort, and anxiety/depression and graded into three levels (level 1 = no problem; level 2 = some/moderate problem; and level 3 = extreme problem). This study will use a Korean estimate weighted-value model of health-related quality of life [24]

The Health Utility Index III (HUI-III) consists of health measures on sight, hearing, speaking, walking, agility, emotion, cognition, and pain, and quality of life values will be calculated by assigning section results to preference score calculation function following the method suggested by Kopec et al. [25].

Lumbar range of movement (ROM) will also be used to objectively assess improvement after treatment. While ROM evaluation is reliable (*r* = 0.94) and valid (*r* = 0.97) [26], it is not highly responsive (effect size 0.1–0.6) [27]. The angle between a perpendicular line and the patient’s lumbar spine will be measured using a goniometer at maximum lumbar flexion, extension, sidebending, and rotation, respectively. If measurement is not possible due to pain, the angle will be recorded as 0°.

#### Cost data investigation

Costs associated with health care may be largely divided into three categories: medical, nonmedical, and lost productivity cost [28–30]. Medical cost covers direct expenses incurred by the use of medical facilities and services (official medical cost) and purchase of health foods and medical devices (unofficial medical cost). Nonmedical cost refers to accompanying costs incidental to medical use such as transportation, patient time, and nursing costs. Lost productivity cost represents economic loss from decreased work capability because of the disease itself or premature death due to disease. Cost data for economic evaluation will be collected through structured survey of medical, nonmedical, and lost productivity cost categories (basic items will be included or excluded according to period).

#### Credibility and expectancy questionnaire

The credibility and expectancy questionnaire will be used to assess treatment expectation on a 9-point Likert scale [31]. Participants will be asked to select an answer to the following questions on their first visit (1 = “not at all”; and 5 = “somewhat”; to 9 = “very much”): “How much do you expect that *Chuna* manual therapy, usual care and combined treatment of *Chuna* with usual care will respectively alleviate your symptoms?”

### Sample size

Determination of study sample size for pCRN: the following assumptions were made for calculation of valid sample size based on previous literature: (1) level of significance (*α*) = 0.05, (2) type 2 error (*β*) = 0.2; with test power set at 80% and (3) referring to NRS difference from prior Korean medicine research using the NRS as the primary measure [32, 33], and average difference and standard deviation (SD) between the three groups specified as 1.5 and 2.5, respectively, by a designated statistician, (4) compliance was set as 80%.

G*Power 3.1.7 was used for sample size calculation, and based on (3) and applied values of mean ± SD, the derived effect size was 0.49. Consequent sample size, as determined by (1) and (2), and applying acquired sample size, was 45 (15 per group).

These results imply that to test the above hypotheses and factoring in a dropout rate of 25%, each group requires a minimum 15 × 100/75 ≒ 20 (subjects), resulting in a total 60 participants.

### Statistical analysis

Intention-to-treat (ITT) analysis will be the primary analysis used for all subjects who receive one or more treatment sessions, and additional per-protocol analysis will be performed in subjects who complete clinical trial participation, excluding dropouts. The primary endpoint will be at 1 week following the end of treatment (7 weeks post random allocation). Continuous data will be analyzed with a paired *t* test for pre-post within-group difference and with an independent *t* test for group comparison between *Chuna* and usual care if normally distributed, and the Wilcoxon rank sum test if not. For comparing between the three groups of *Chuna* with usual care, *Chuna*, and usual care, if continuous data satisfies the normality test, analysis of variance (ANOVA) will be used to test between-group difference, and the Kruskal-Wallis test if it does not. *Chuna* with usual care versus *Chuna*, and *Chuna* with usual care versus usual care will be analyzed using Duncan’s post-hoc test. Post-hoc analysis of non-normally distributed data will use the Wilcoxon rank sum test with significance level adjusted by the Bonferroni test. Categorical data will be analyzed using the chi-square test or Fisher’s exact test. A paired *t* test will be used in pre-post within-group differences by time point. Secondary analyses will include analysis of covariance (ANCOVA) adjusting for statistically significant between-group differences in baseline outcomes or covariant factors at baseline, and subgroup analyses by LBP disease differentiation (nonspecific LBP, lumbar intervertebral disc herniation, and spinal stenosis), pain duration (subacute, chronic, and hyperchronic), and main site of pain (LBP worse than leg pain, and LBP less than leg pain) to explore the feasibility of a large, multicenter, pragmatic RCT.

All statistical analysis will use a two-tailed test as a principle, and significance level will be set at 5%. In addition, last observation carried forward (LOCF) will be conducted to handle missing data as it is currently widely used in clinical trials.

### Economic evaluation

Economic evaluation will be performed to assess cost-effectiveness of the three groups. The primary economic endpoint will be cost per quality-adjusted life year (QALY) gained, and secondary economic endpoints will cover such effectiveness parameters as cost per NRS. The primary analysis period will be 14 weeks and, if further estimation is needed, secondary analysis using regression-model or decision-modelling analysis to extrapolate cost and effect beyond the follow-up period will be performed. Treatment costs related to the clinical trial will be calculated by multiplying number of treatment sessions with unit cost, and unit costs will use national health insurance and medical institution cost data. Estimates of quality of life for QALYs will use the EQ-5D as the main assessment variable in the area under the curve method [34]. In the case that total time horizon is at least 12 months, the unit cost will be standardized using the Korean monetary unit (won) value as of 2013 and applying a discount rate of 5% in accordance with Korean HIRA economic evaluation guidelines. The analysis perspective of this study will be social and, in baseline analysis, representative values (e.g., average) of study parameters will be used. All available distribution and representative values of parameter estimates will be applied in probabilistic sensitivity analysis.

### Data collection and management

Paper CRFs will be used and a standard operating procedure (SOP) for CRF entry will be prepared. Investigators and the contract research organization (CRO) at the lead study site (Pusan National University Korean Medicine Hospital) will educate outcome assessors and investigators at each site regarding CRF entry, electronic data conversion, and SOP. Double data entry will be allowed in CRF outcome measurement data, with data entered once at each study site, and once at the lead organization and CRO, respectively. After confirming whether electronic data conversion using double entry is accurate, data access will be blocked to all researchers except statisticians.

### Adverse events

Physicians will monitor and record any unexpected or unintended patient reaction to *Chuna*, usual care, or its combination at each visit. Adverse events (AEs) associated with *Chuna* will include, but not be limited to, AEs anticipated from previous reports of manual therapy and will stay open to all possibilities taking into consideration other potential, unknown AEs. Physicians will rate the causal relationship between each treatment method and AE occurrence on a 6-point scale (1 = definitely related; 2 = probably related; 3 = possibly related; 4 = probably not related; 5 = definitely not related; and 6 = unknown), and all AEs will be categorized into three levels using the Spilker classification (mild (1) = not needing additional intervention, nor significantly inhibiting to the normal lifestyle (function) of the participant; moderate (2) = significantly inhibiting to the normal lifestyle (function) of the participant, and may need additional intervention, recovering afterwards; and severe (3) = severe AE requiring intensive intervention, and leaving sequelae). In the case of “serious adverse event (SAE)” during study duration, unblinding is permissible and the relevant Institutional Review Board (IRB) and main study site (Pusan National University Korean Medicine Hospital) will be informed and will decide whether the trial will continue or be terminated. In the event that a subject suffers direct injury associated with the clinical trial, the subject may receive appropriate medical action as seen fit by the subinvestigator, and damage compensation will follow prespecified clinical research insurance clauses. All study participants will be provided with an emergency contact number to the principal investigator or subinvestigator for any questions, medical issues or research-related diseases during the study and accordingly receive requested services.

### Data monitoring and safety monitoring

This study is a pilot study with no interim analysis, and a data monitoring committee was not organized as no SAEs regarding *Chuna* or usual care not previously reported are expected to occur.

Safety assessment will mainly assess frequency of AEs, including all SAEs. AE reports will summarize the number and percentage of participants who experienced AEs, and will include categorized information on body region of AE manifestation. Other data (e.g., severity, causal relationship with treatment) will be included in safety monitoring reports.

An outside CRO will be entrusted with safety and data monitoring. CRO monitoring will consist of three sessions in the following order: initiative monitoring at first subject enrollment, one midterm monitoring during the clinical trial, and conclusive monitoring upon completion of clinical trial. Participant safety data and CRF and source documents will be reviewed and crosschecked to confirm participant safety and data integrity.

### Stopping rules

The trial will be stopped in the case that the principle investigator regards that there is unacceptable risk of SAE(s) within group(s).

## Discussion

Usual care was selected as the choice of comparator in this study for investigation of the effectiveness and safety of *Chuna* manual therapy. Various national and international clinical practice guidelines and systematic reviews were consulted in discussion of usual care contents for LBP. While current guidelines indicate the benefits of conservative treatment [5], the evidence for individual conservative treatment methods and opinions on what should constitute standard care of LBP are somewhat inconsistent [4–6]. Moreover, recent reports state that the effect of acetaminophen, which is recommended for first-line use in LBP guidelines [4–6], is unclear [35]. Therefore, in this study we opted to use the most frequently used LBP treatments in Korea as opposed to an evidence-based selection. Korea has a unitary government-run national health insurance system which aims to cover the entire South Korean population and all medical institutions. This insurance system reimburses HIRA-approved medical costs for the greater majority of diseases including LBP, and the HIRA database contains extensive information on medication, medical services, and diagnosis [17]. For this study, we extracted the most frequently used treatment types in patients with a main diagnosis code of LBP from the 2011 HIRA national patients’ sample (NPS) data, and provided this list to usual care providers for reference in patient treatment in an effort to secure validity in usual care treatment selection.

While the updated Cochrane review on spinal manipulative therapies for chronic LBP covers many study designs similar to the current study comparing spinal manipulation with other treatments, such as usual care, or as an add-on treatment to other treatments, such as usual care alone, evidence for the effect of spinal manipulative therapy is not conclusive [36]. However, as *Chuna* manual therapy and the spinal manipulative therapies included in the Cochrane review hold distinct differences, clinical effects may also be disparate. Meanwhile, a 2013 systematic review of RCTs on *Chuna* published in the Korean *Chuna* literature states that the two RCTs related to LBP both support a significant effect of *Chuna*, but that they are limited by qualitative issues and small sample size [37]. Therefore, it can be inferred from these evaluations that further rigorous clinical studies on *Chuna*, a Korean spinal manipulative therapy, are in order.

The aim of this pCRN study is to examine the comparative clinical effectiveness of *Chuna*, a Korean spinal manual therapy, through comparison of *Chuna* manual therapy and usual care treatments most frequently used in Korea, and investigate whether the combined treatment of *Chuna* manual therapy and usual care, which is reflective of, and more frequently encountered, than singular treatment of *Chuna* or usual care in real-world clinical practice, is more effective than single treatment. While this pilot study may fail to reach a definite conclusion due to current sample size, it will still hold relevance as one of the first well-designed, rigorous RCTs on *Chuna* manual therapy for nonacute LBP patients, and provide preliminary effectiveness and safety monitoring results for future large-scale, multicenter RCTs.

### Trial status

Participant recruitment is anticipated to be from March to August 2016, and study completion to be reported in December 2016. The full-scale, multicenter RCT (CRN study) is expected to be initiated in 2017 after analysis of pCRN data.

## Additional files

Additional file 1. SPIRIT (Standard Protocol Items: Recommendations for Interventional Trials) 2013 Checklist. Description of data: populated SPIRIT (Standard Protocol Items: Recommendations for Interventional Trials) 2013 Checklist: recommended items to address in a clinical trial protocol and related documents. (DOC 123 kb)
Additional file 2. Trial committee organization and contributions and role. Description of data: trial committee organization and contributions and role in accordance with SPIRIT (Standard Protocol Items: Recommendations for Interventional Trials) 2013 Checklist. (DOCX 29 kb)
Additional file 3.
*Chuna* manual therapy technique application method. Description of data: technical details of *Chuna* manual therapy with photos. (DOCX 2822 kb)

## Abbreviations

AE: Adverse event
ANCOVA: Analysis of covariance
ANOVA: Analysis of variance
CMT: *Chuna* manual therapy
CRF: Case Report Form
CRIS: Clinical Research Information Service
CRO: Contract research organization
EQ-5D: EuroQol-5 dimensions
HIRA: Health Insurance Review and Assessment
HUI-III: Health Utility Index III
IRB: Institutional Review Board
KMD: Korean medicine doctor
LBP: Low back pain
LOCF: Last observation carried forward
NPS: National patients’ sample
NRS: Numeric Rating Scale
ODI: Oswestry Disability Index
pCRN: Pilot *Chuna* Research Network
PGIC: Patient Global Impression of Change
QALY: Quality-adjusted life years
RCT: Randomized controlled trial
ROM: Range of movement
SAE: Serious adverse event
SD: Standard deviation
SMT: Spinal manipulative therapy
SOP: Standard operating procedure
SPIRIT: Standard Protocol Items: Recommendations for Interventional Trials
SR: Systematic review
UC: Usual care
