# Supplementary material for: Comparative effectiveness and cost-effectiveness of Chuna manual therapy versus conventional usual care for nonacute low back pain: study protocol for a pilot multicenter, pragmatic randomized controlled trial (pCRN study)
Source: Trials. 2017 Jan 17;18:26. doi: 10.1186/s13063-016-1756-8 (PMC5240424; doi:10.1186/s13063-016-1756-8)
Supplement: Additional file 2. — Trial committee organization and contributions and role. Description of data: trial committee organization and contributions and role in accordance with SPIRIT (Standard Protocol Items: Recommendations for Interventional Trials) 2013 Checklist. (DOCX 29 kb) [file 13063_2016_1756_MOESM2_ESM.docx]

**Principal investigator (PI) and research physicians at main study site**

**Organization: Pusan National University Korean Medicine Hospital**

**Contributions and role:** Principal investigator, original study design: Byung-Cheul Shin (Email: [drshinbc@pusan.ac.kr](mailto:drshinbc@pusan.ac.kr))

Organization of Steering committee and member appointment: Byung-Cheul Shin, Eui-Hyoung Hwang, Kwang-Ho Heo, In Heo, Man-Suk Hwang, and Byung-Jun Kim

Communication and exchange of opinion with PI at each site

Preparation of IRB documents and CRF

Trial management (randomized allocation management, AE data collection at each site, participant enrollment supervision, study site inspection and visits, budget allocation and management)

Cooperation with CRO in data collection, quality control, monitoring, and analysis

**Steering committee (SC)**

**Organization and role:** All authors of this manuscript

**Contributions:** Protocol revision and decision on final protocol

Organization of Trial Management Committee and member appointment

Designation of participant recruitment study sites

Inspection of study progress, and decision on protocol revision, if needed

Determination of study result publication timing and method

Decision on authorship in accordance with Authorship eligibility guidelines

**Trial Management Committee**

**Organization:** PI and investigators at each clinical trial participant enrollment site

**Organization and role:** Submission and obtaining study protocol approval from relevant IRB of each study site

Clinical trial execution following protocol (e.g. participant recruitment, enrollment, data collection, CRF entry)

Collection and report of AEs
