# Supplementary material for: Comparative effectiveness and cost-effectiveness of Chuna manual therapy versus conventional usual care for nonacute low back pain: study protocol for a pilot multicenter, pragmatic randomized controlled trial (pCRN study)
Source: Trials. 2017 Jan 17;18:26. doi: 10.1186/s13063-016-1756-8 (PMC5240424; doi:10.1186/s13063-016-1756-8)
Supplement: Additional file 3. — Chuna manual therapy technique application method. Description of data: technical details of Chuna manual therapy with photos. (DOCX 2822 kb) [file 13063_2016_1756_MOESM3_ESM.docx]

**1. [Mandatory technique] Spine flexion distraction (flexion) [38, 39, 41]**

1) Overview

| Patient position | Lies prone |
| --- | --- |
| Physician position | Stands next to the pelvic part of the Chuna table |
| Main hand | Contacts the spinous process of the vertebra superior to the target dysfunctional intervertebral disc with the heel of the hand |
| Supporting hand | Presses the table handle to lower the pelvic part of the Chuna table |
| Procedure | -Applied about 4 times per spinal vertebra  -One application should take about 4 seconds |

2) Application method


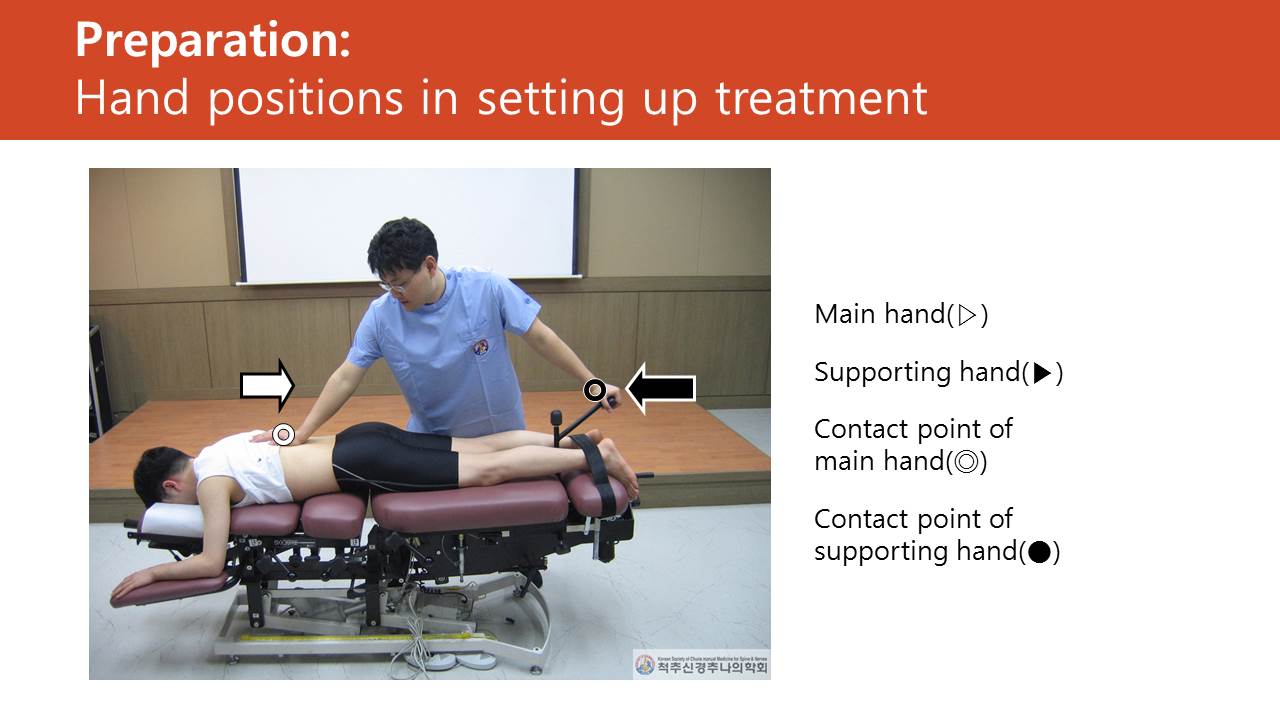

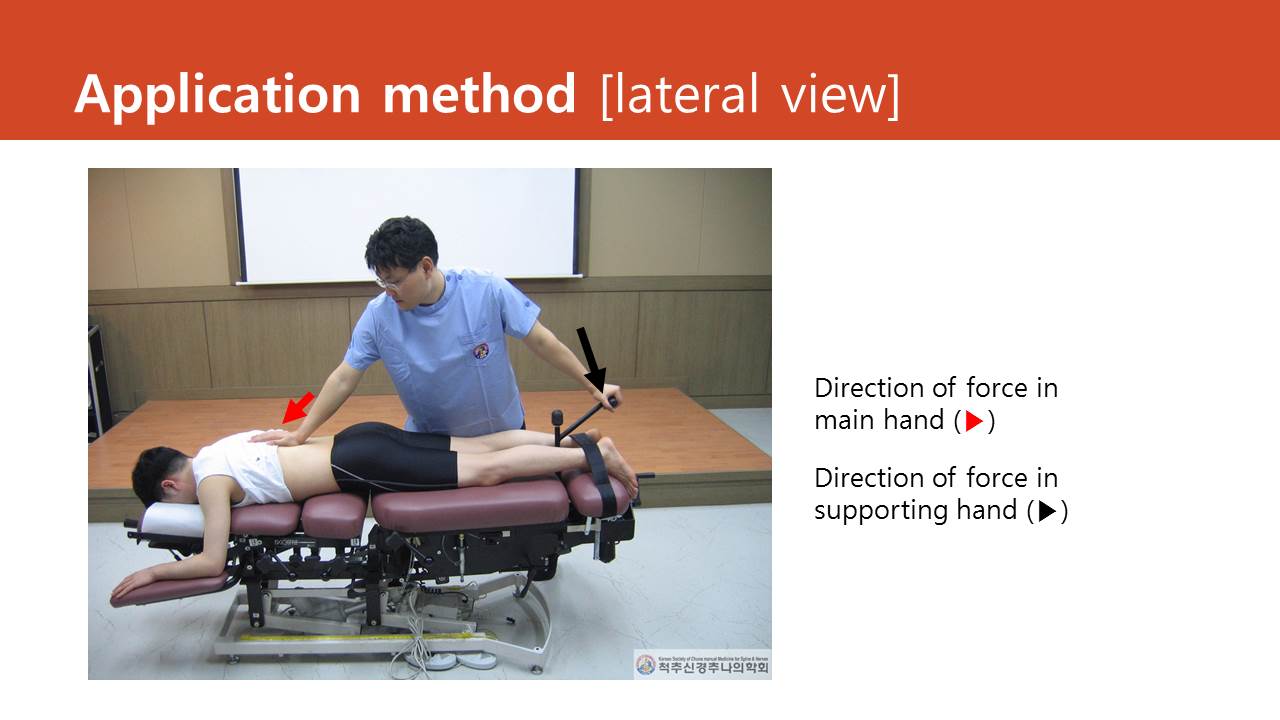

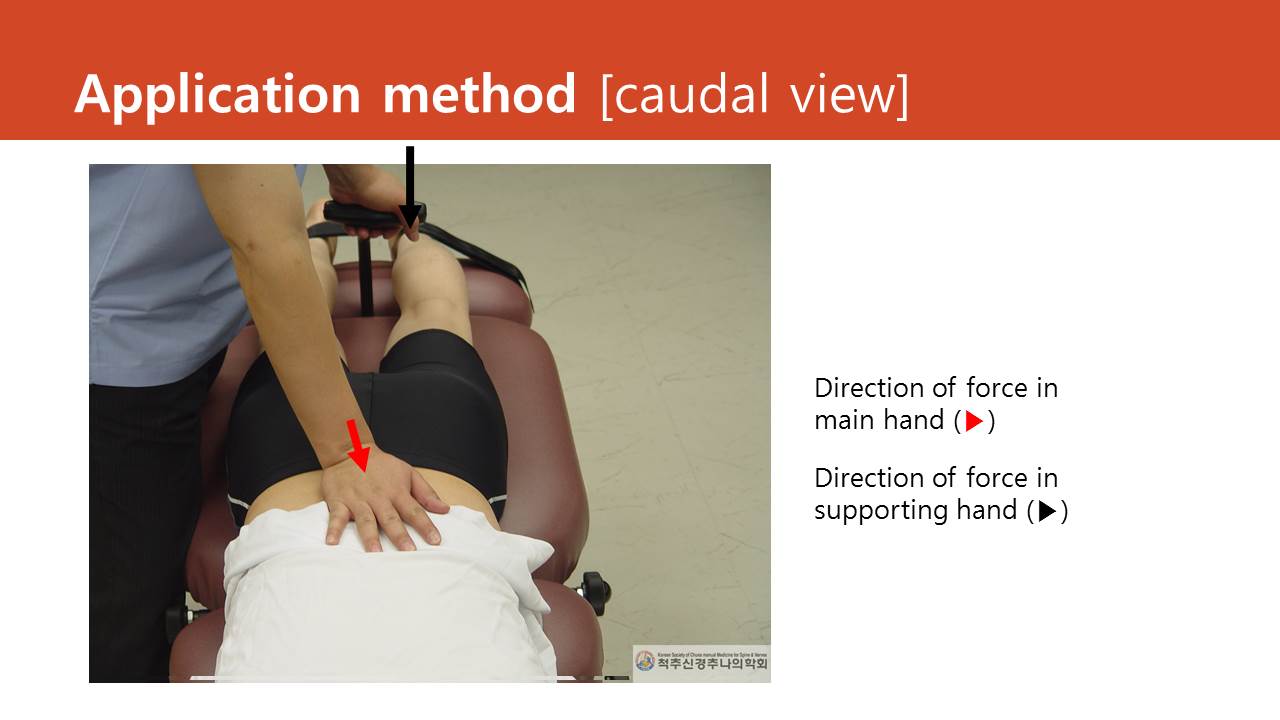

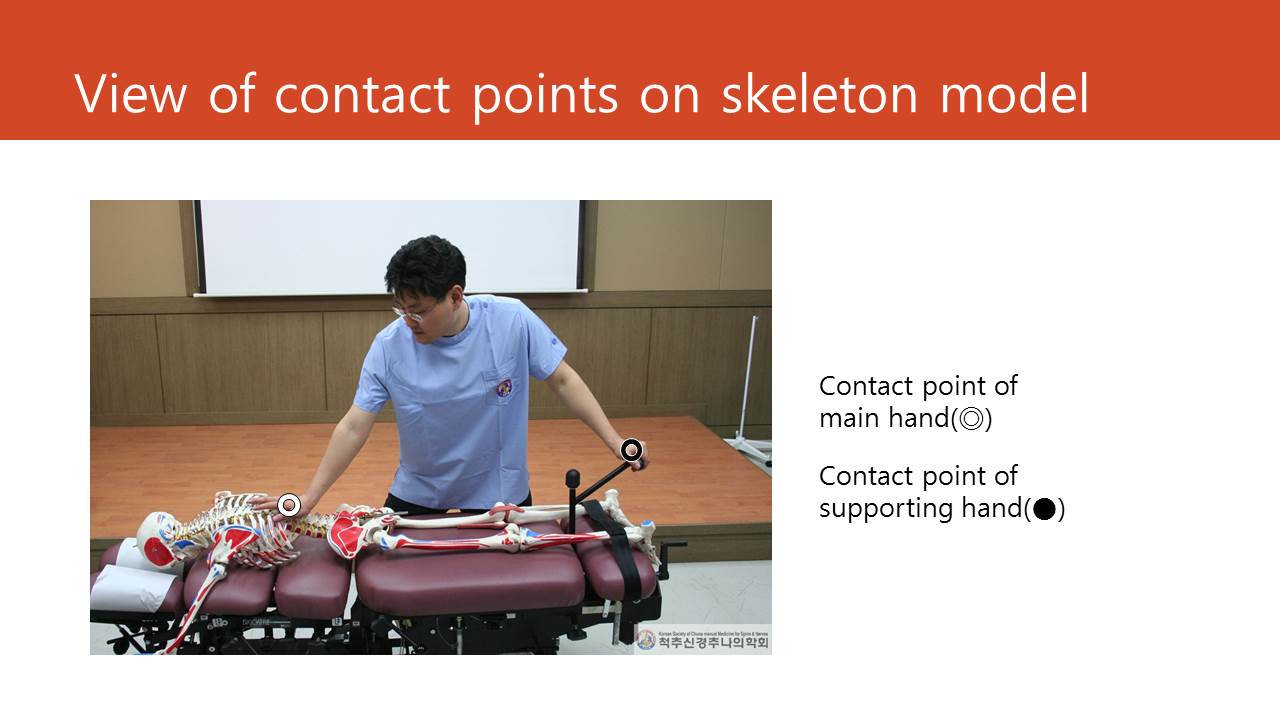


**2. [Selective technique] Spinal flexion distraction (sidebending) [38, 39, 41]**

1) Overview

| Patient position | Lies prone |
| --- | --- |
| Physician position | Stands next to the pelvic part of the Chuna table |
| Main hand | Contacts the spinous process of the vertebra superior to the target dysfunctional intervertebral disc between the thumb (end) and 2^nd^ finger proximal interphalangeal joint (PIP) to limit movement |
| Supporting hand | Holds the table handle to maneuver the pelvic part of the Chuna table to the left or right |
| Procedure | -Applied about 4~8 times per spinal vertebra  -One application should take about 4 seconds |

2) Application method


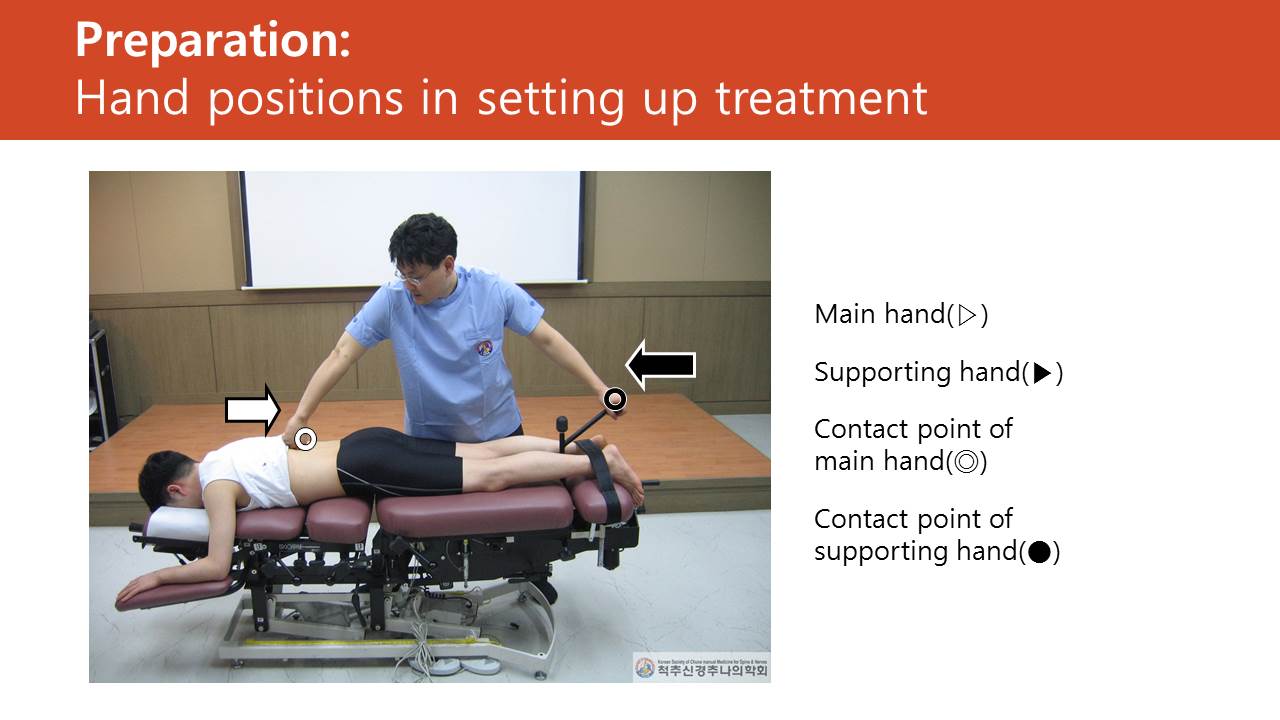

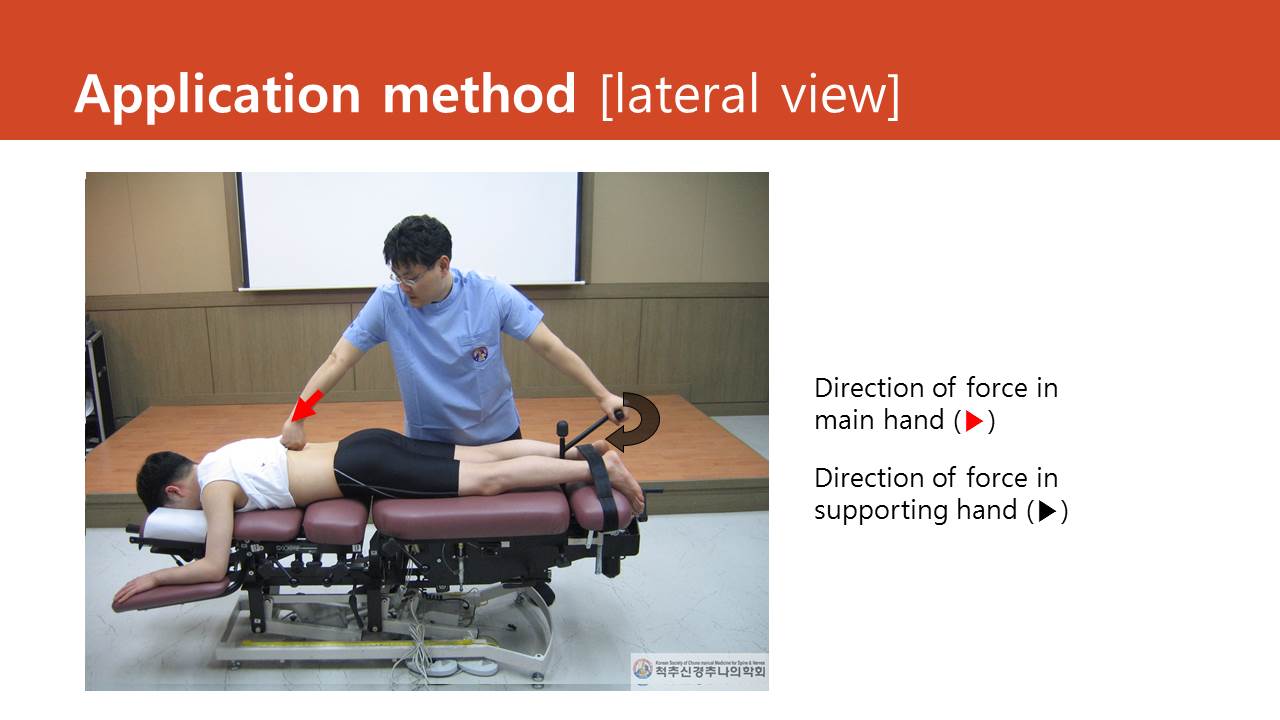

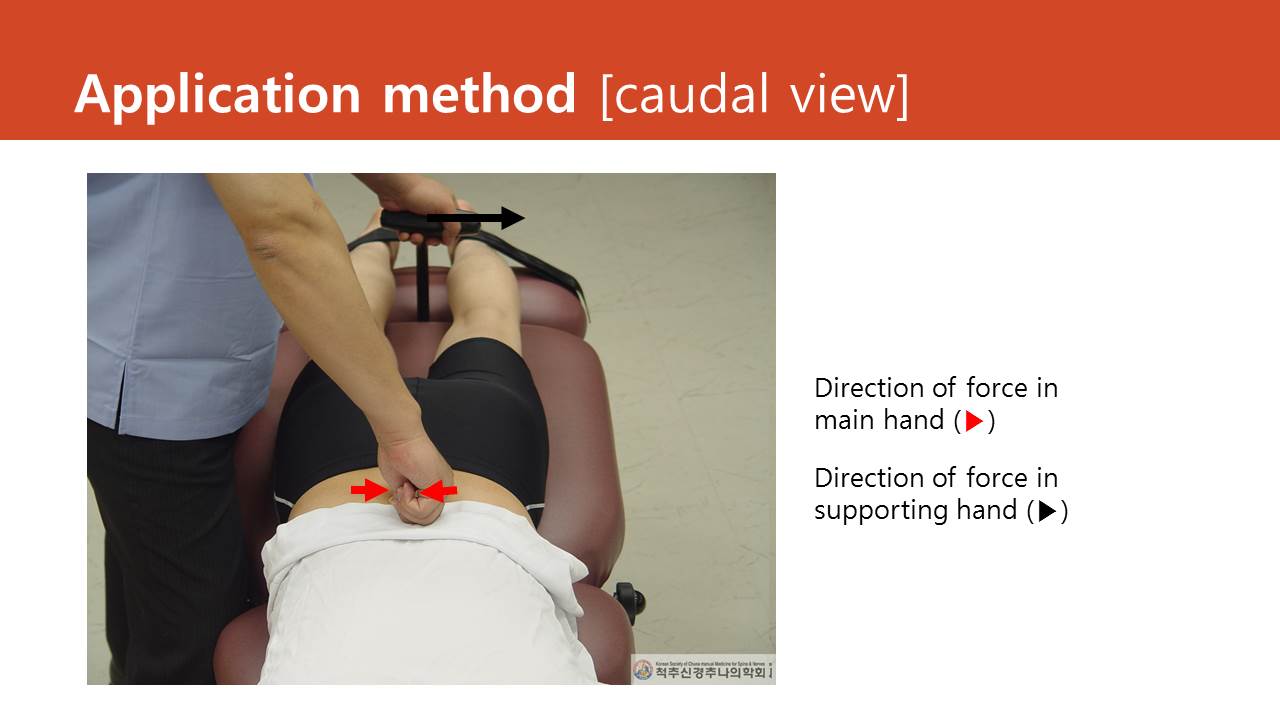

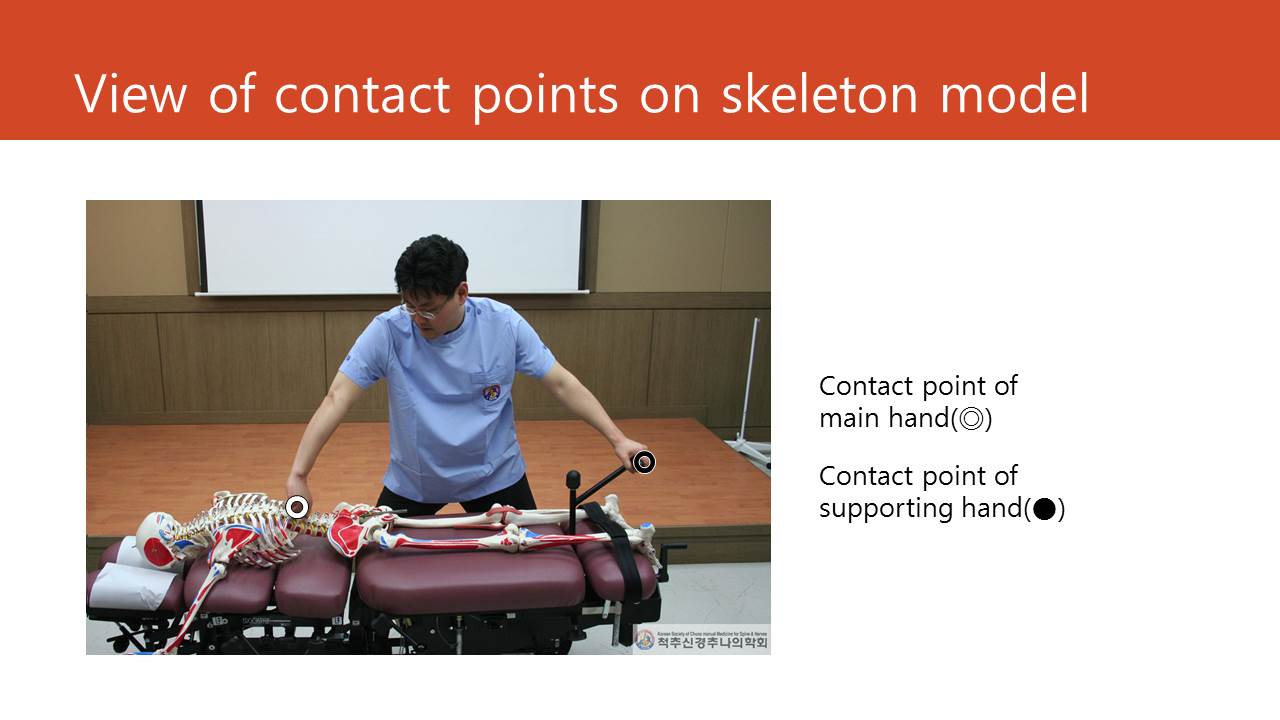


**3. [Selective technique] Spinal flexion distraction (circumduction) [38, 39, 41]**

1) Overview

| Patient position | Lies prone |
| --- | --- |
| Physician position | Stands next to the pelvic part of the Chuna table |
| Main hand | Contacts the spinous process of the vertebra superior to the target dysfunctional intervertebral disc between the thumb (end) and 2^nd^ finger proximal interphalangeal joint (PIP) to limit movement |
| Supporting hand | Holds the table handle to move the pelvic part of the Chuna table laterally, then into flexion distraction, finally returning to the original position |
| Procedure | -Applied about 4 times per spinal vertebra  -One application should take about 4 seconds |

2) Application method


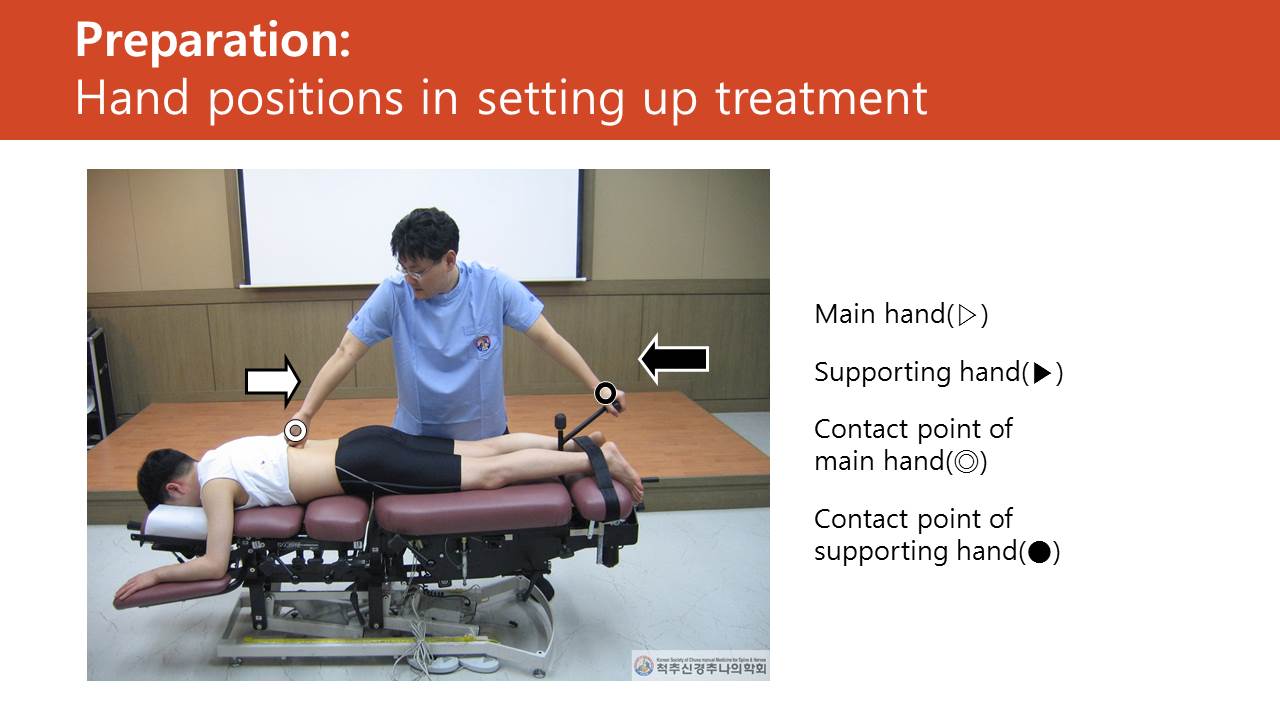

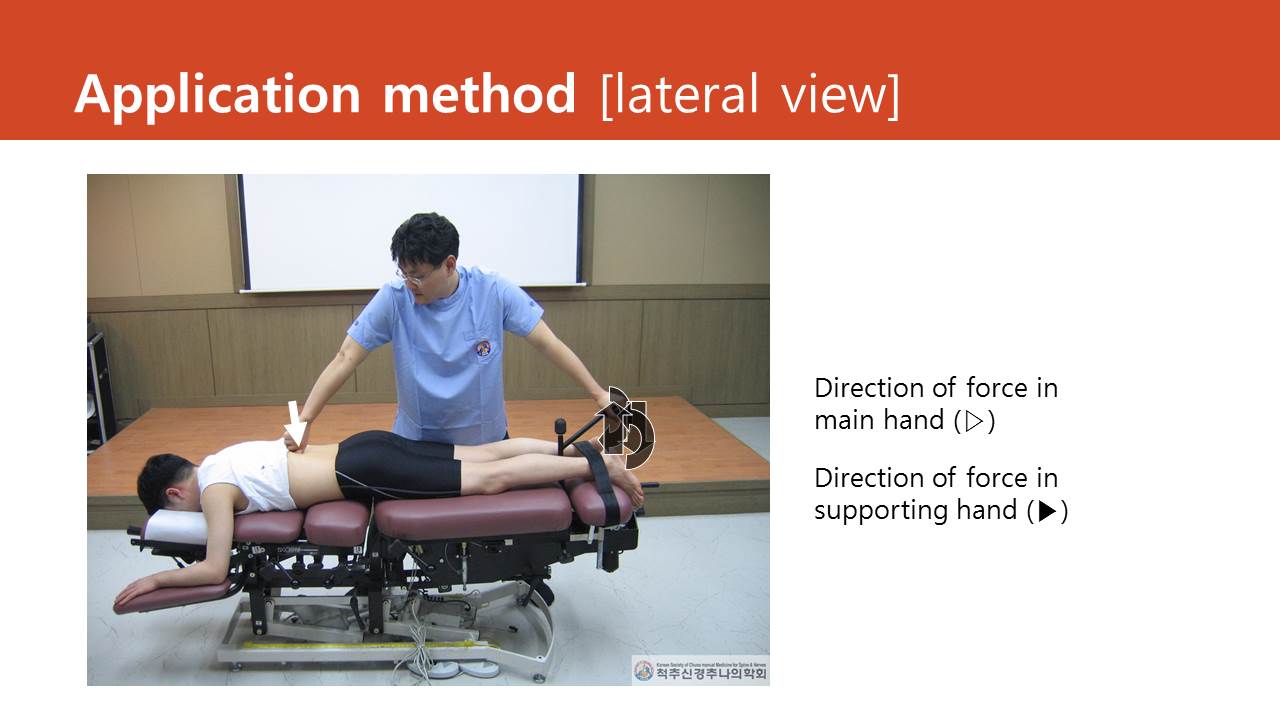

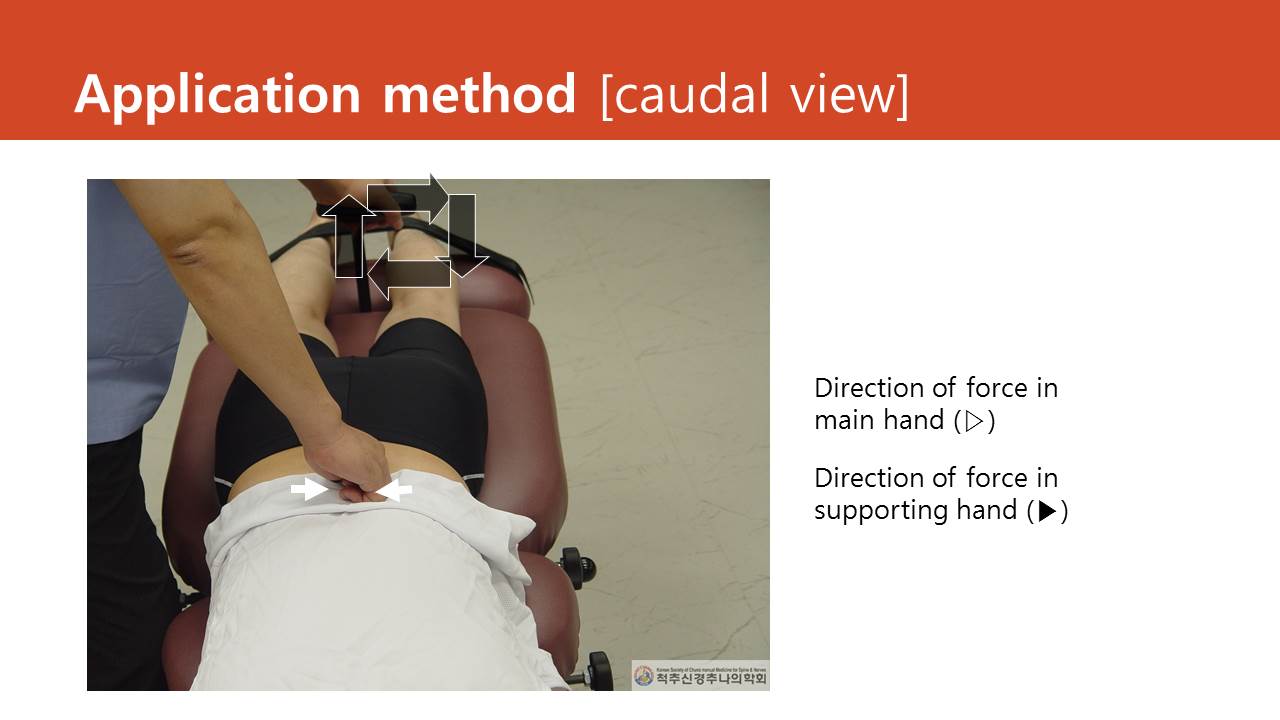

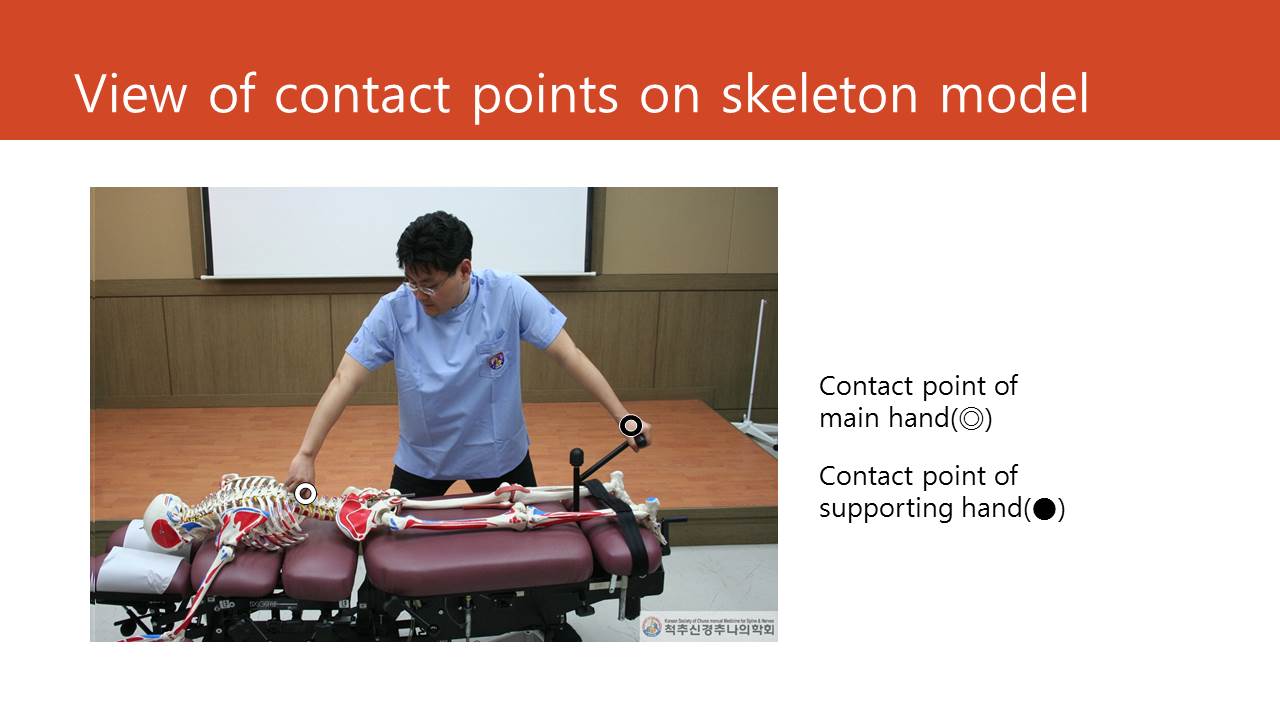


**4. [Mandatory technique] Sidelying lumbar extension dysfunction correction technique [38-40]**

1) Overview

| Dysfunction | Extension, rotation/ipsilateral sidebending (ERS) dysfunction |
| --- | --- |
| Patient position | Lies in sidelying position with the rotated side of the vertebra facing down |
| Physician position | Stands in fencing stance facing the patient |
| Main hand | Contacts the patient’s ilium with the forearm of the caudal hand, and corrects with a sudden short force directed anterior superior |
| Supporting hand | Contacts the patient’s axillary area with the forearm of the cephalad hand, and maintains the patient’s torso in a rotated position |

2) Application method


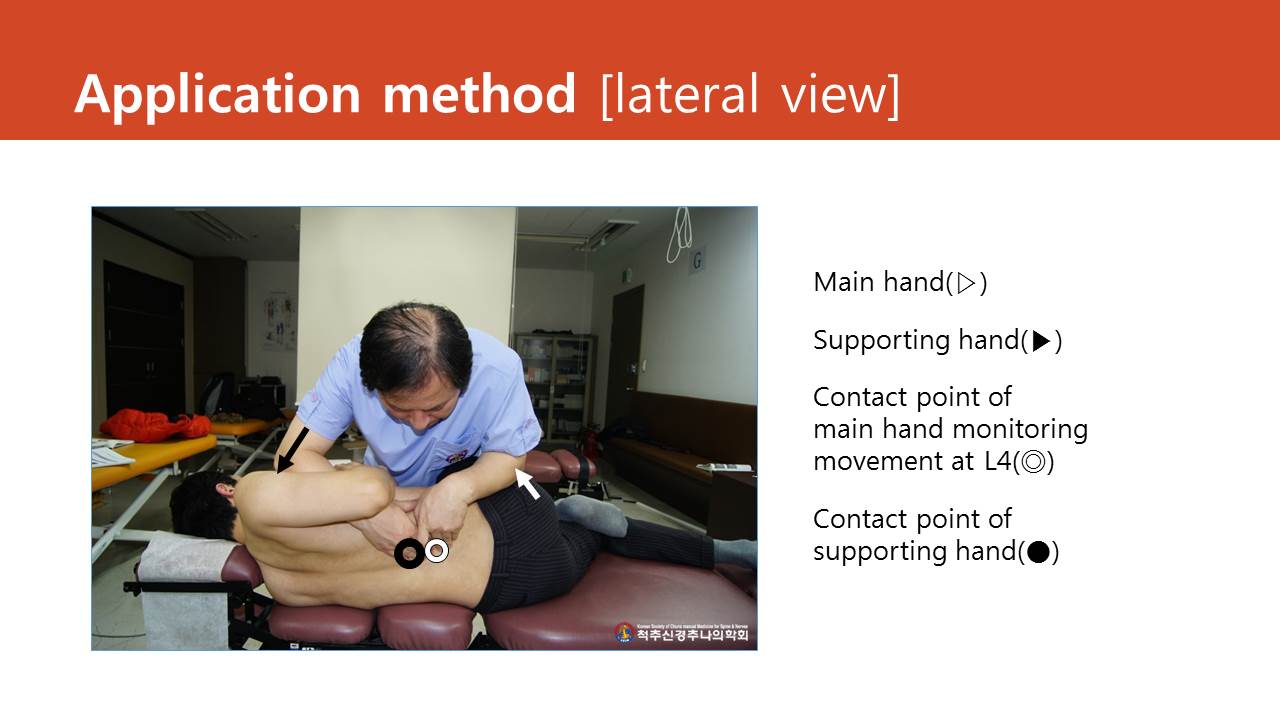


**5. [Mandatory technique] Sidelying lumbar flexion dysfunction correction technique [38-40]**

1) Overview

| Dysfunction | Flexion, rotation/ipsilateral sidebending (FRS) dysfunction |
| --- | --- |
| Patient position | Lies in sidelying position with the rotated side of the vertebra facing down |
| Physician position | Stands in fencing stance facing the patient |
| Main hand | Contacts the patient’s ilium with the forearm of the caudal hand, and corrects with a sudden short force directed anterior superior |
| Supporting hand | Contacts the patient’s axillary area with the forearm of the cephalad hand, and maintains the patient’s torso in a rotated position |

2) Application method


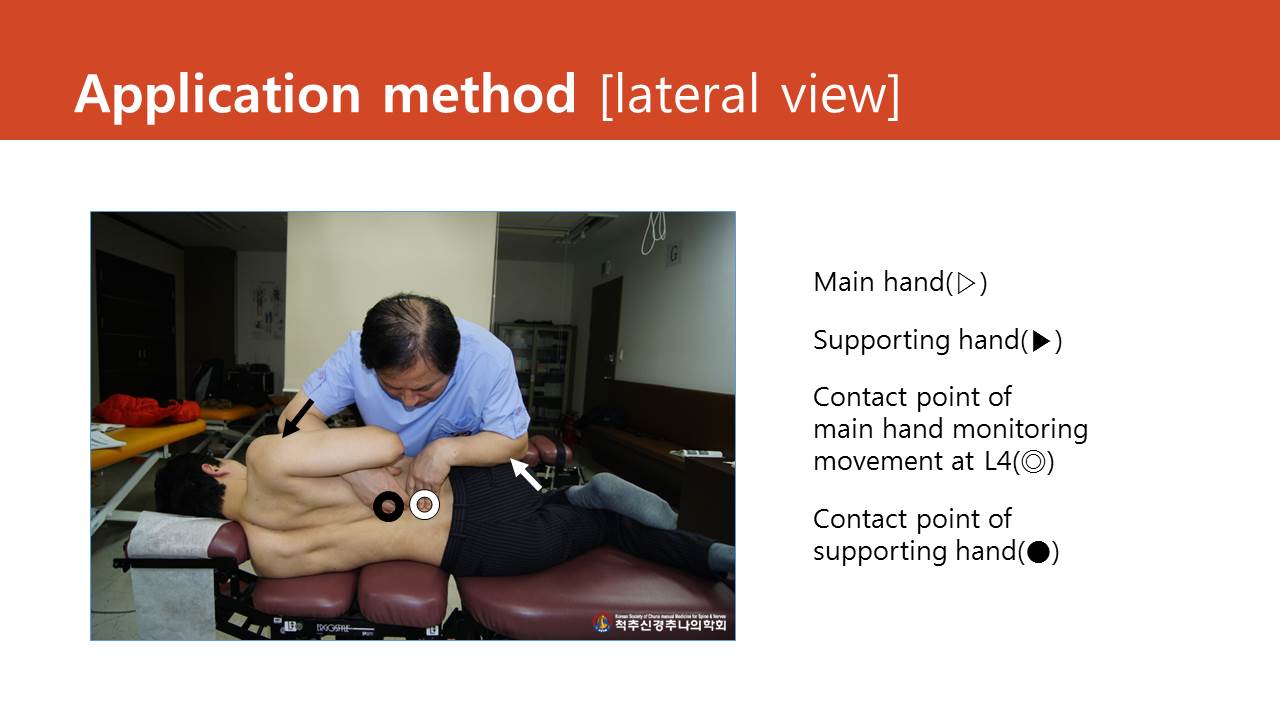


**6. [Selective technique] Iliopsoas fascial Chuna [38-40]**

1) Overview

| Patient position | Lies prone at the caudal end of the bed/Chuna table with the proximal part of the patient’s thighs contacting the bed/Chuna table (The patient flexes the contralateral hip and knee, and uses both hands to pull the knee toward the chest until the low back area contacts the bed/Chuna table) |
| --- | --- |
| Main hand | Contacts the quadriceps femoris muscle portion superior to the patella |
| Supporting hand | Contacts the ipsilateral anterior superior iliac spine (ASIS) |
| Direction of force | The physician extends the patient’s ipsilateral leg to check the restriction barrier of the iliopsoas muscle/fascia, then backs up to midrange and has the patient breathe in and hold their breath, and implement isometric contraction in the direction of hip flexion using the iliopsoas muscle/fascia (with 20% of maximum force) while the physician applies resistance of the same force. The physician instructs the patient to breath out after 6~7 seconds releasing force, then repeats 3~4 times |
| Post-application | 4-second resting phase, followed by stretching of the iliopsoas muscle/fascia for about 8 seconds |

2) Application method


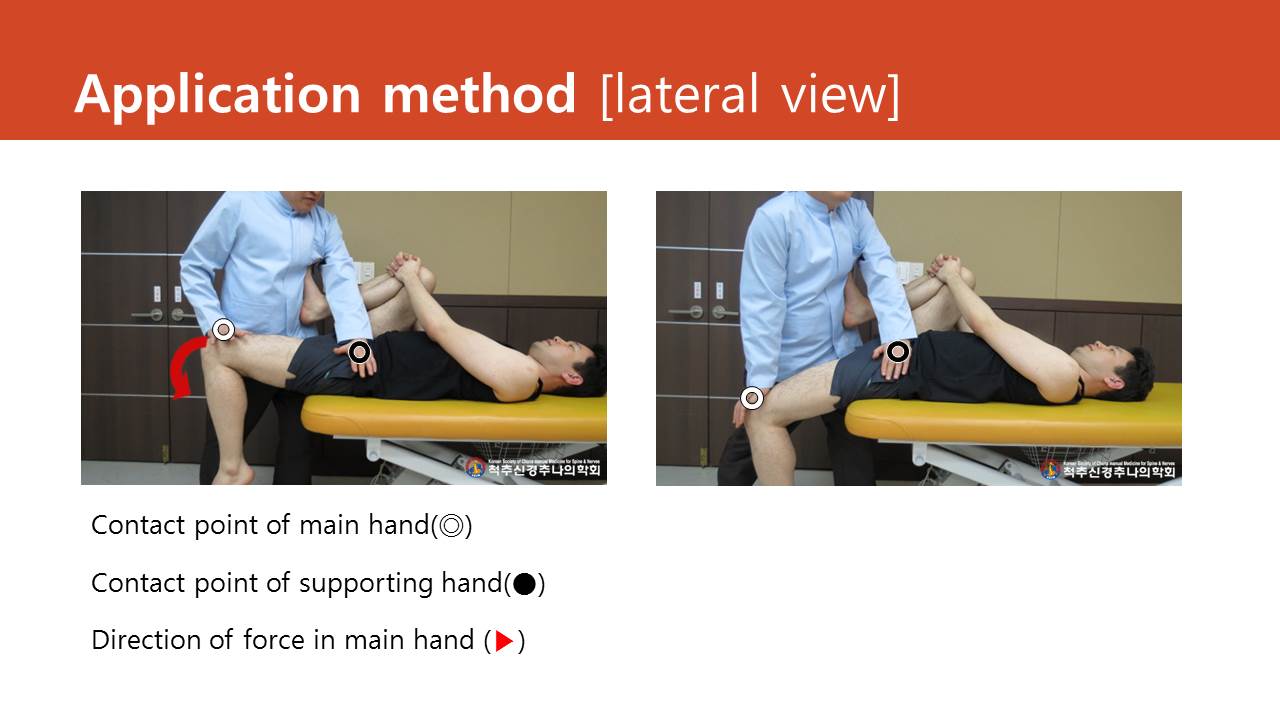


**7. [Mandatory technique] Prone iliac anterior rotation dysfunction correction technique [38, 39, 41]**

1) Overview

| Indication | Iliac anterior rotation dysfunction (previously referred to as ‘anterior superior ilium’) |
| --- | --- |
| Patient position | Lies prone |
| Physician position | Stands in fencing stance on the ipsilateral side of the anterior rotation dysfunctional ilium (anterior superior ilium) |
| Main hand | Contacts the ipsilateral ischial tuberosity with the pisiform bone area of the cephalad hand |
| Supporting hand | Supports the wrist of the main hand with the caudal hand |

2) Application method


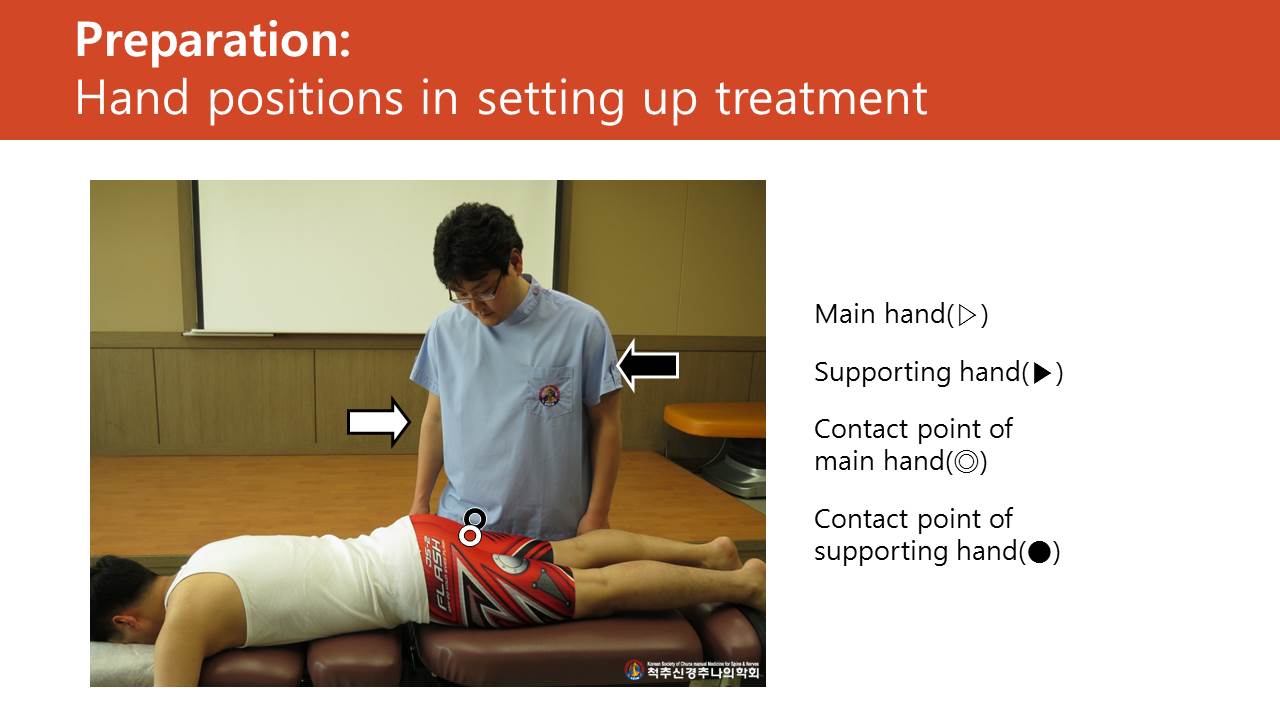

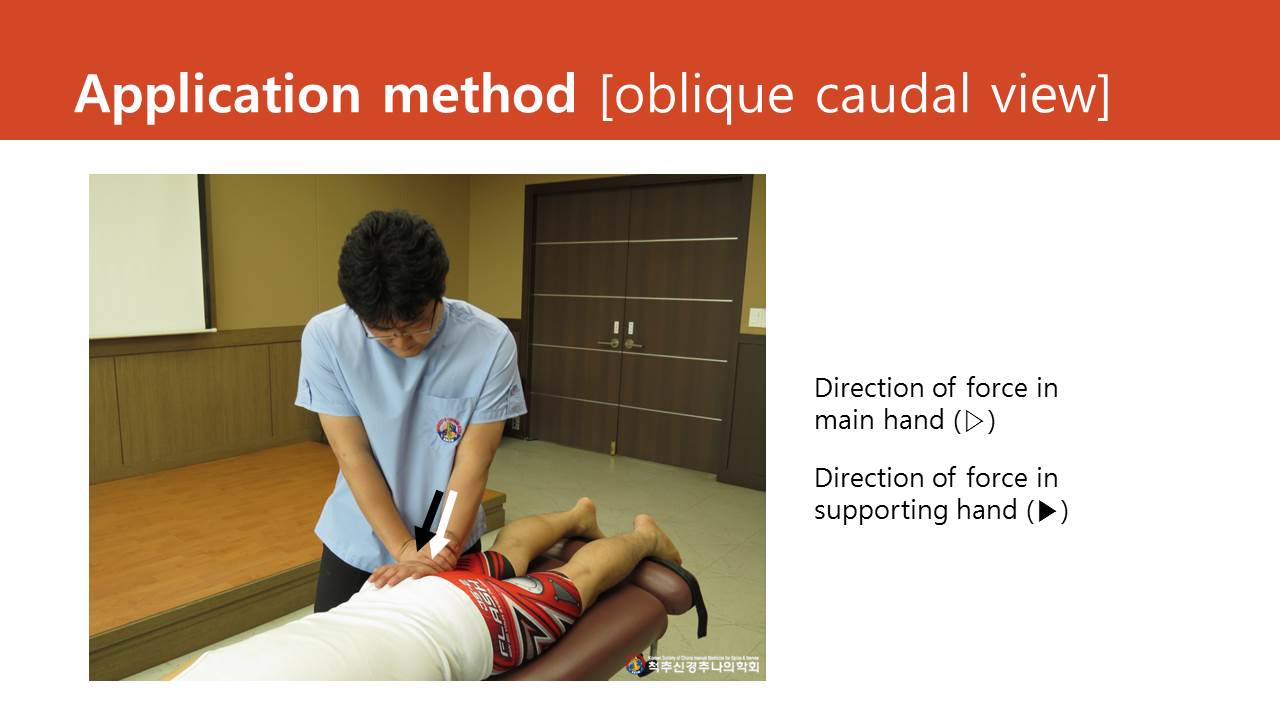

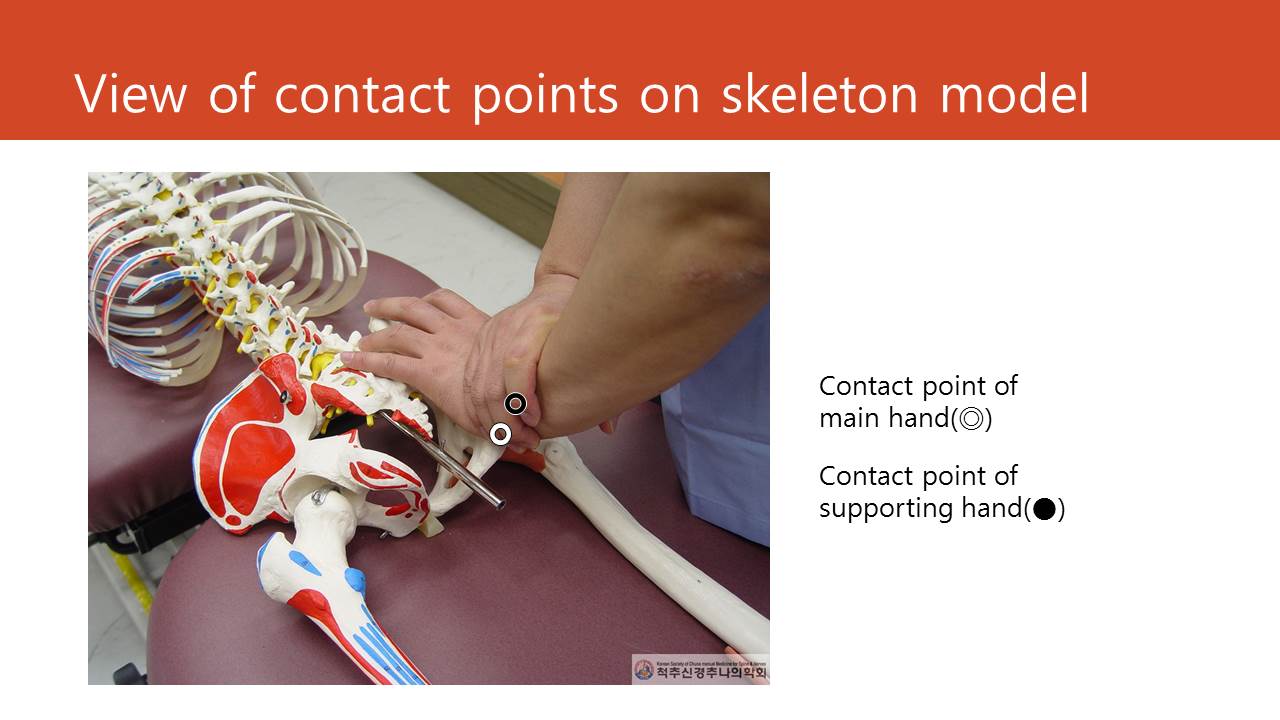


**8. [Mandatory technique] Prone iliac posterior rotation/sacral sidebending dysfunction correction technique [38, 39, 41]**

1) Overview

| Indication | Iliac posterior rotation dysfunction (previously referred to as ‘posterior inferior ilium’) |
| --- | --- |
| Patient position | Lies prone |
| Physician position | Stands contralateral to the patient |
| Main hand | Contacts the ipsilateral posterior superior iliac spine (PSIS) with the heel of the cephalad hand of the posterior rotation dysfunctional ilium (posterior inferior ilium) |
| Supporting hand | Contacts the contralateral ischial tuberosity using the metacarpophalangeal joint (MCP) of the 2^nd^ finger of the caudal hand |

2) Application method


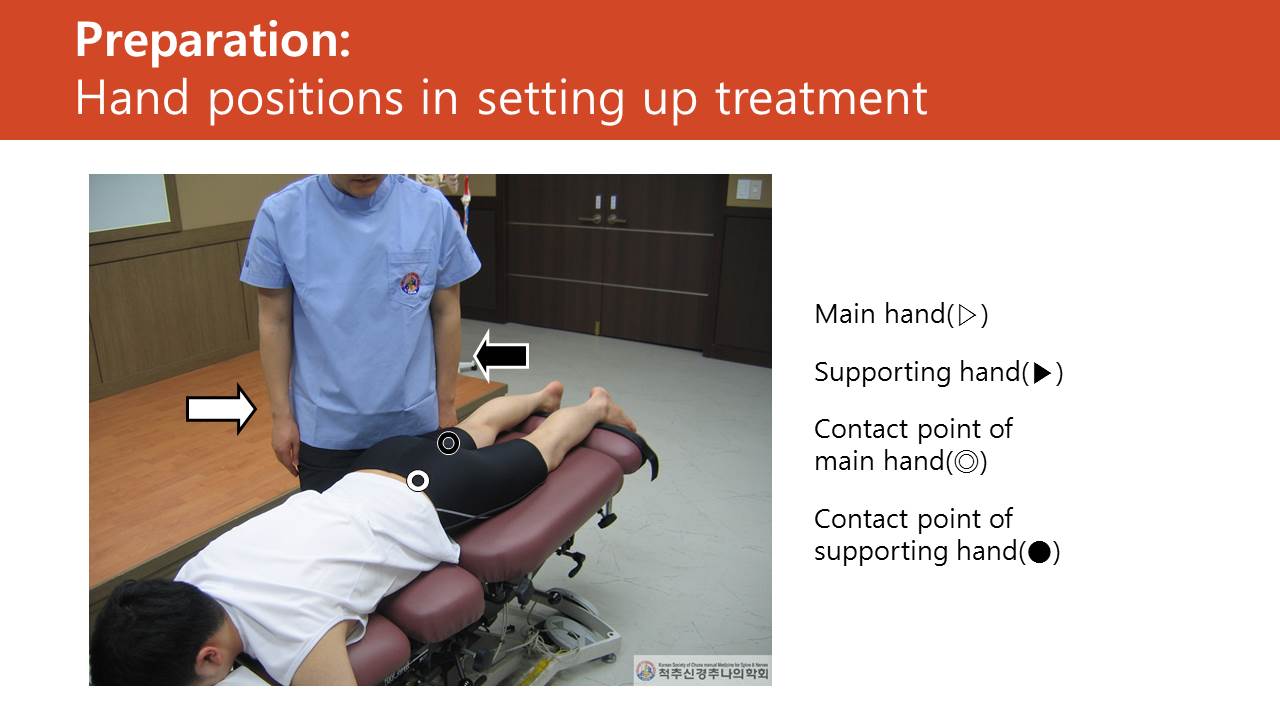

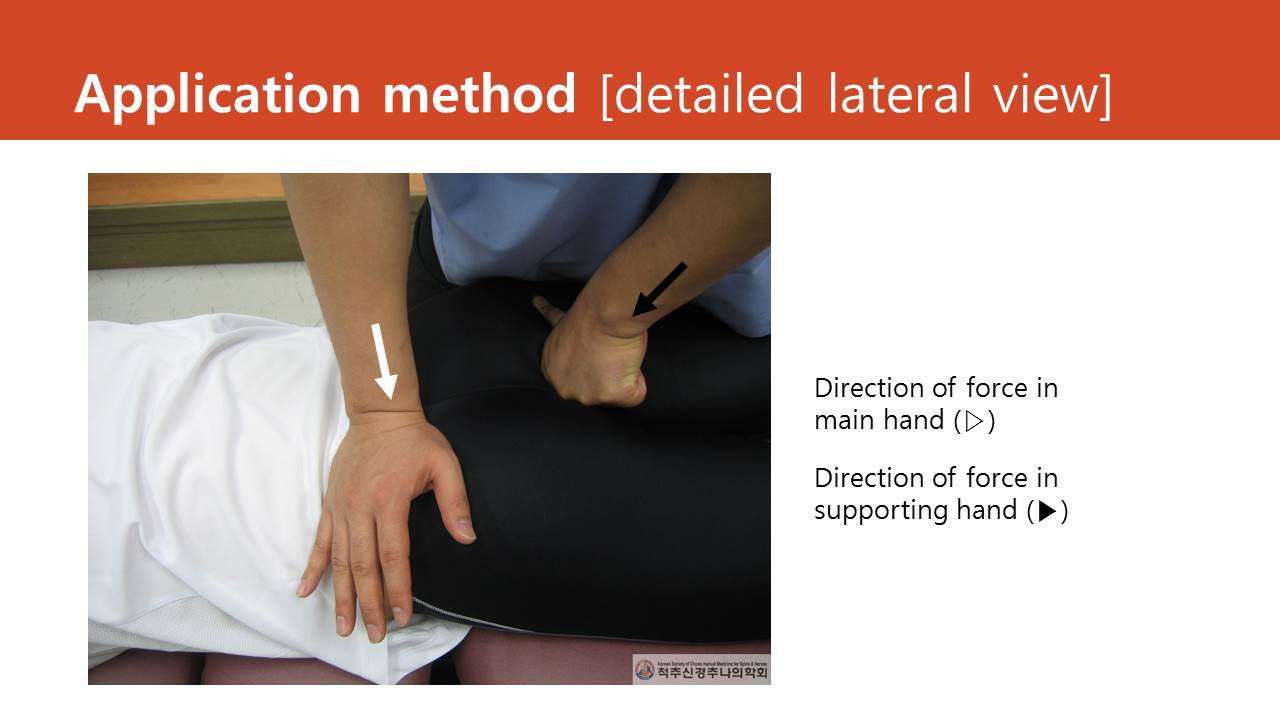

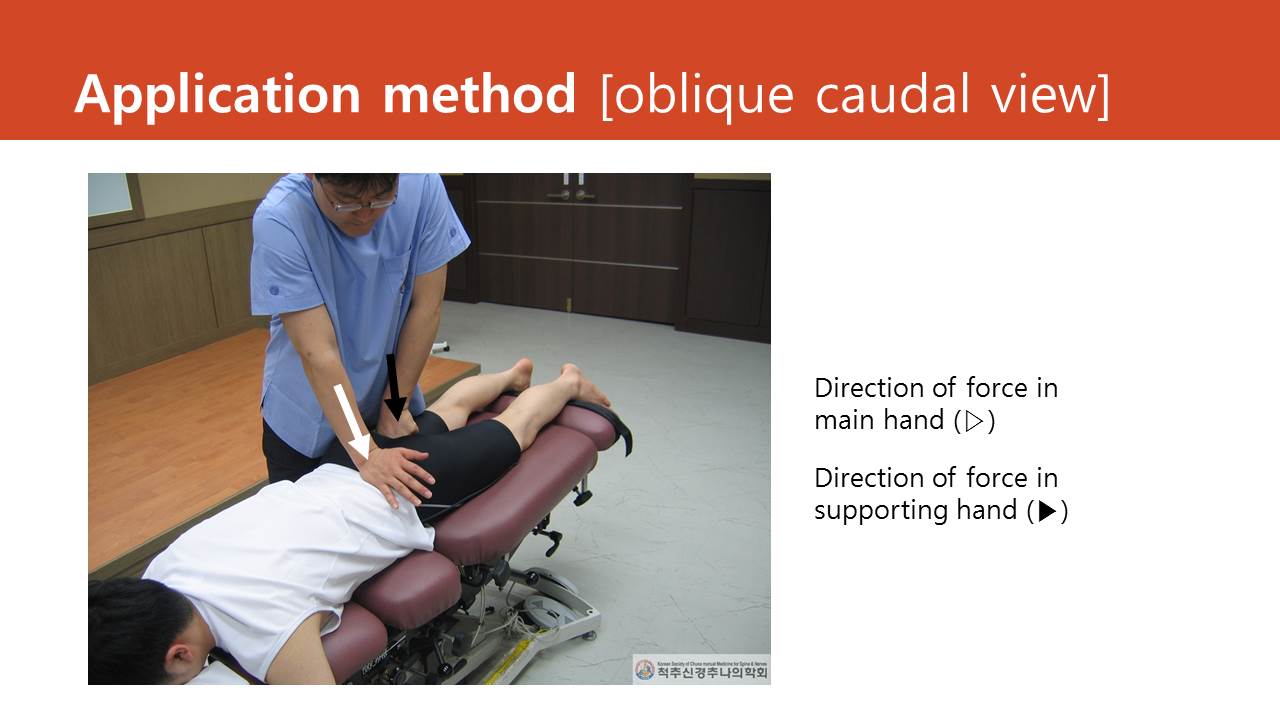

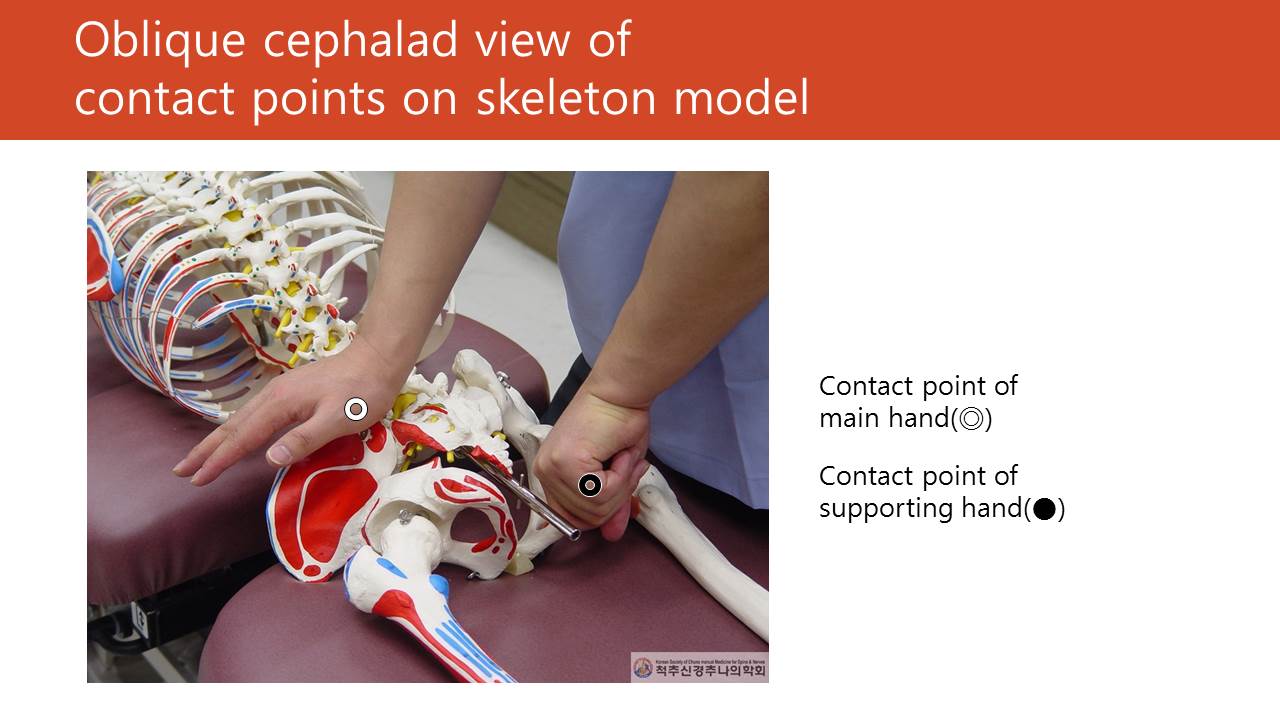


**9. [Mandatory technique] Prone leg raise iliac dysfunction correction technique [38, 39, 41]**

1) Overview

| Indication | Iliac posterior rotation dysfunction (previously referred to as ‘posterior inferior ilium’) |
| --- | --- |
| Patient position | Lies prone |
| Physician position | Stands ipsilateral to the patient |
| Main hand | Contacts the ipsilateral posterior superior iliac spine (PSIS) with the pisiform bone area |
| Supporting hand | Raises the ipsilateral leg superior to the patellar joint contacting the medial side |

2) Application method


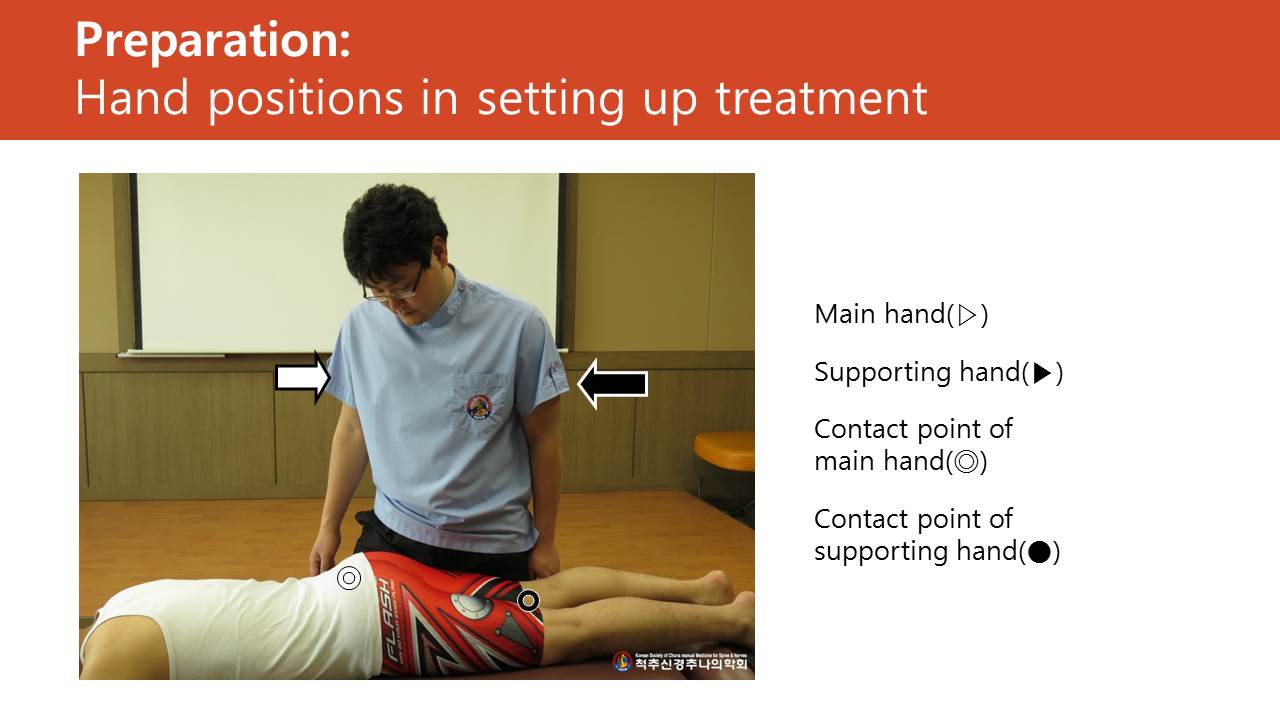

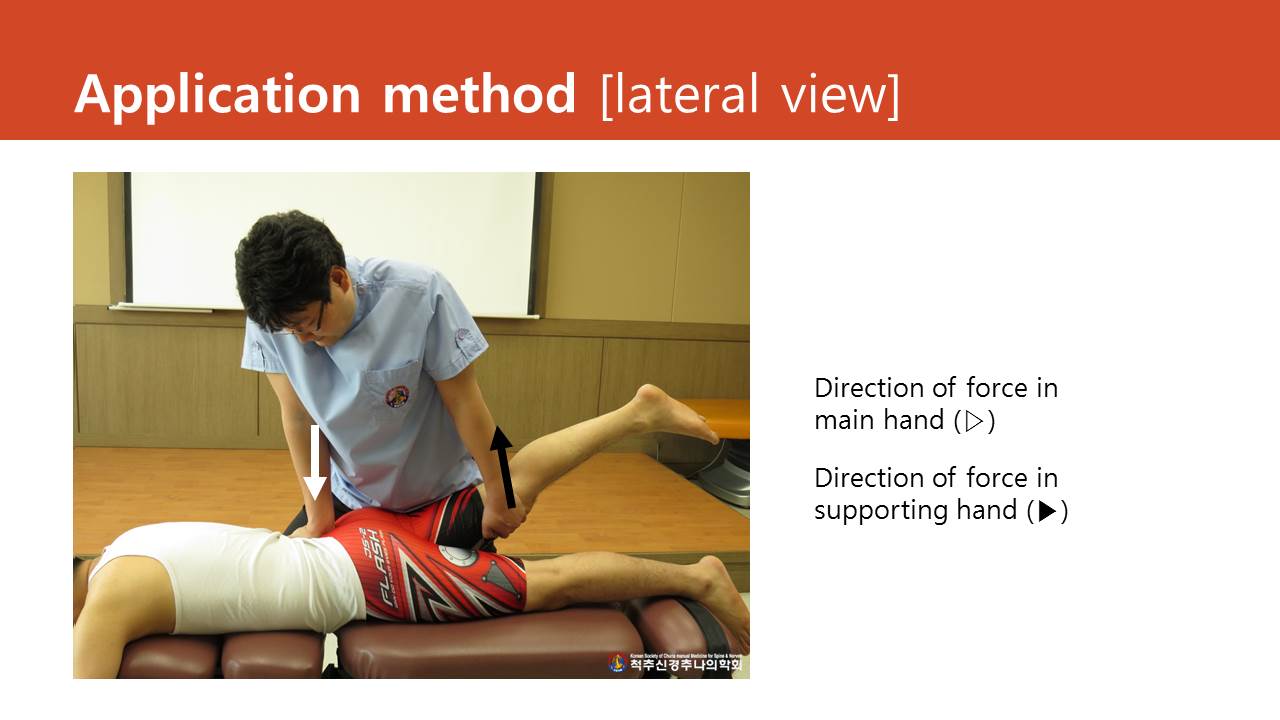

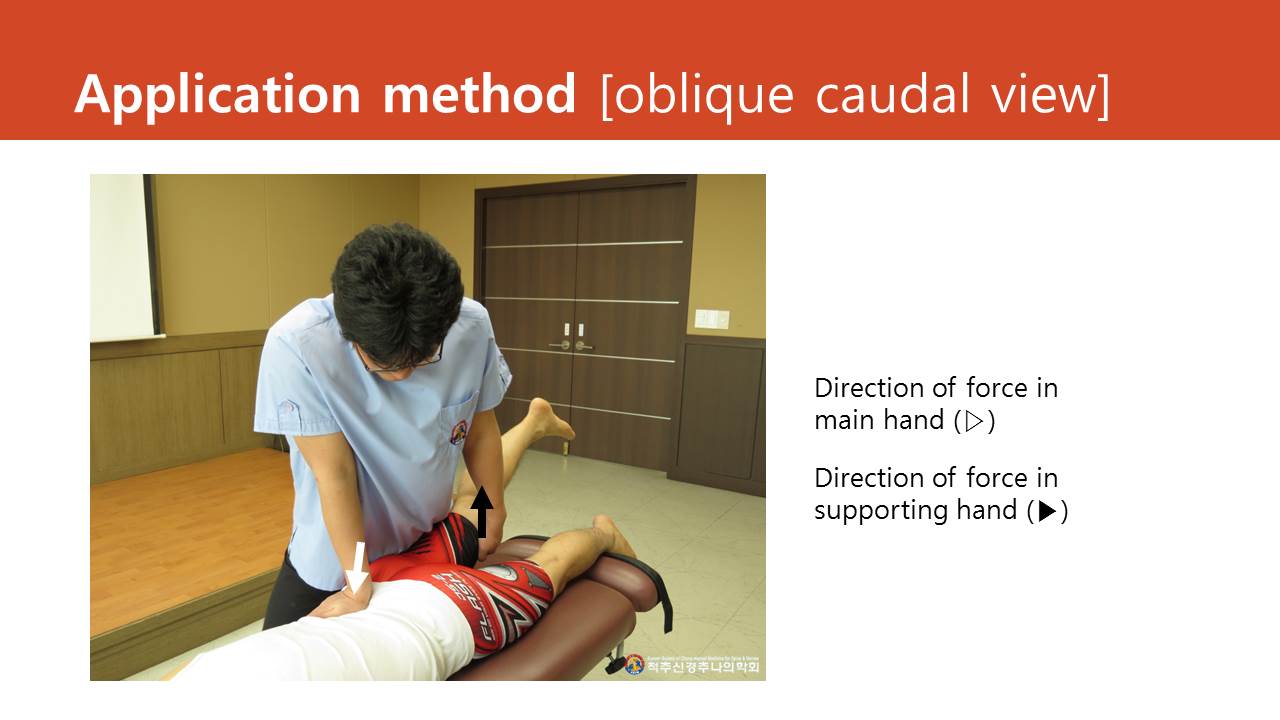

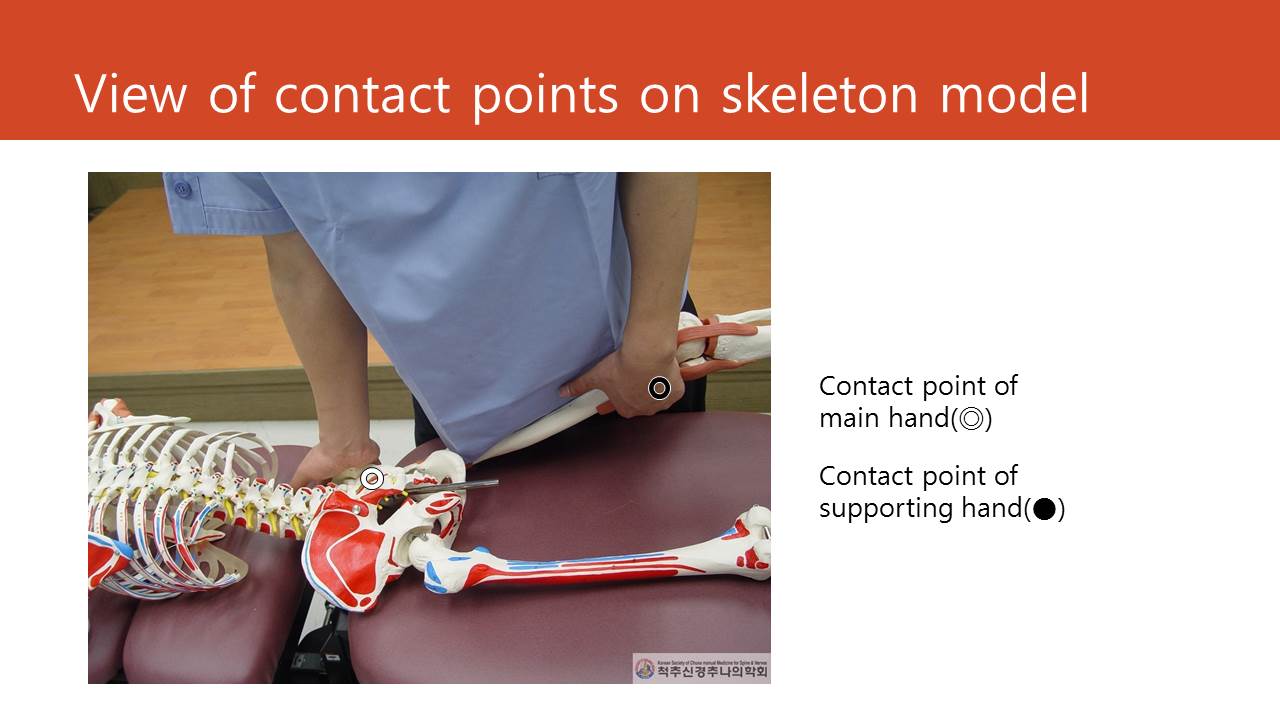


**10. [Mandatory technique] Prone inflare-outflare dysfunction correction technique [38, 39, 41]**

1) Overview

| Indications | -Inflare (narrowed) (previously referred to as ‘lateral ilium’)  -Outflare (widened) (previously referred to as ‘medial ilium’) |
| --- | --- |
| Patient position | Lies prone |
| Physician position | Stands ipsilateral to the dysfunctional ilium |
| Main hand | Contacts the ipsilateral posterior superior iliac spine (PSIS) of the dysfunctional ilium with the pisiform bone area of the cephalad hand |
| Supporting hand | Contacts the ipsilateral ischial tuberosity using the metacarpophalangeal joint (MCP) of the 2^nd^ finger of the caudal hand |

2) Application method


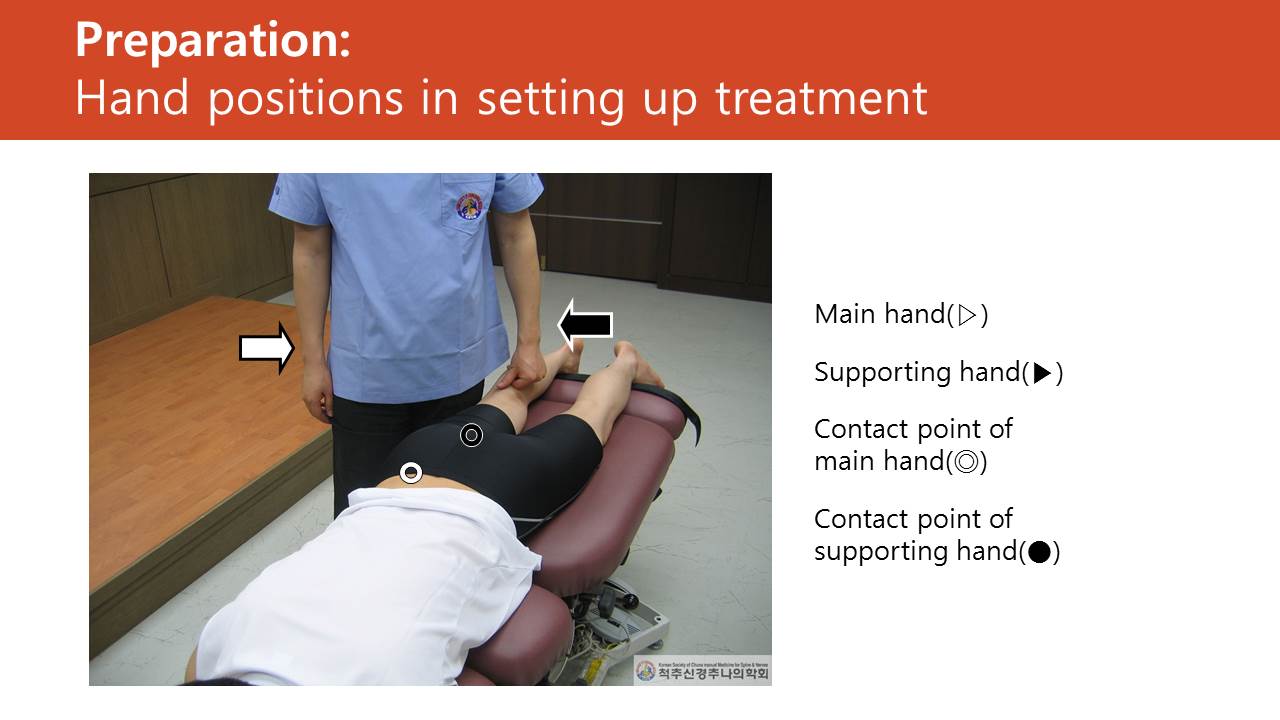

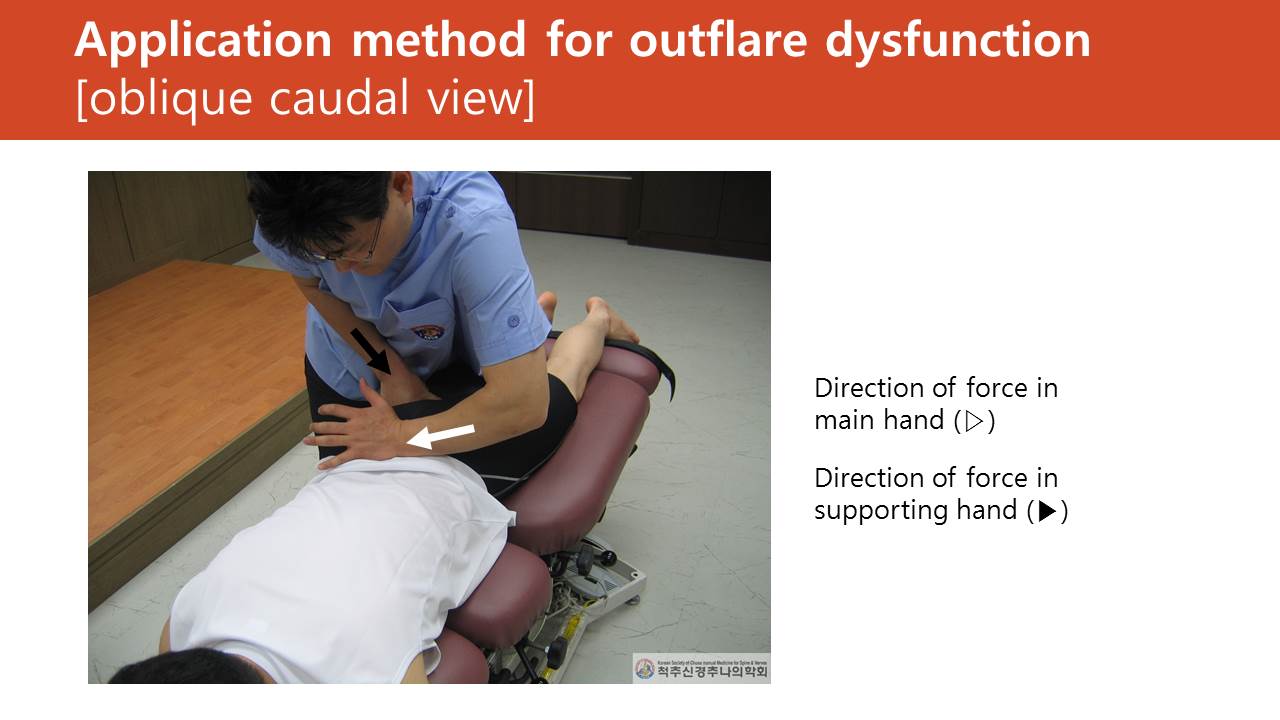

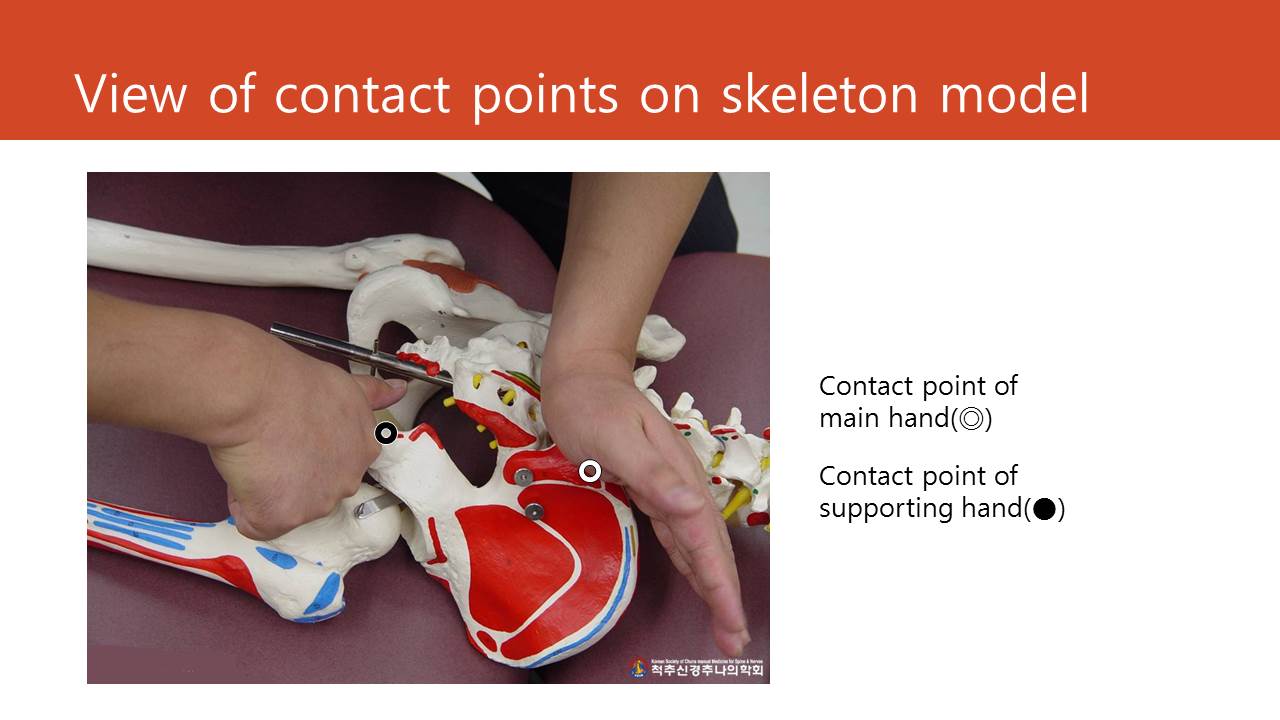

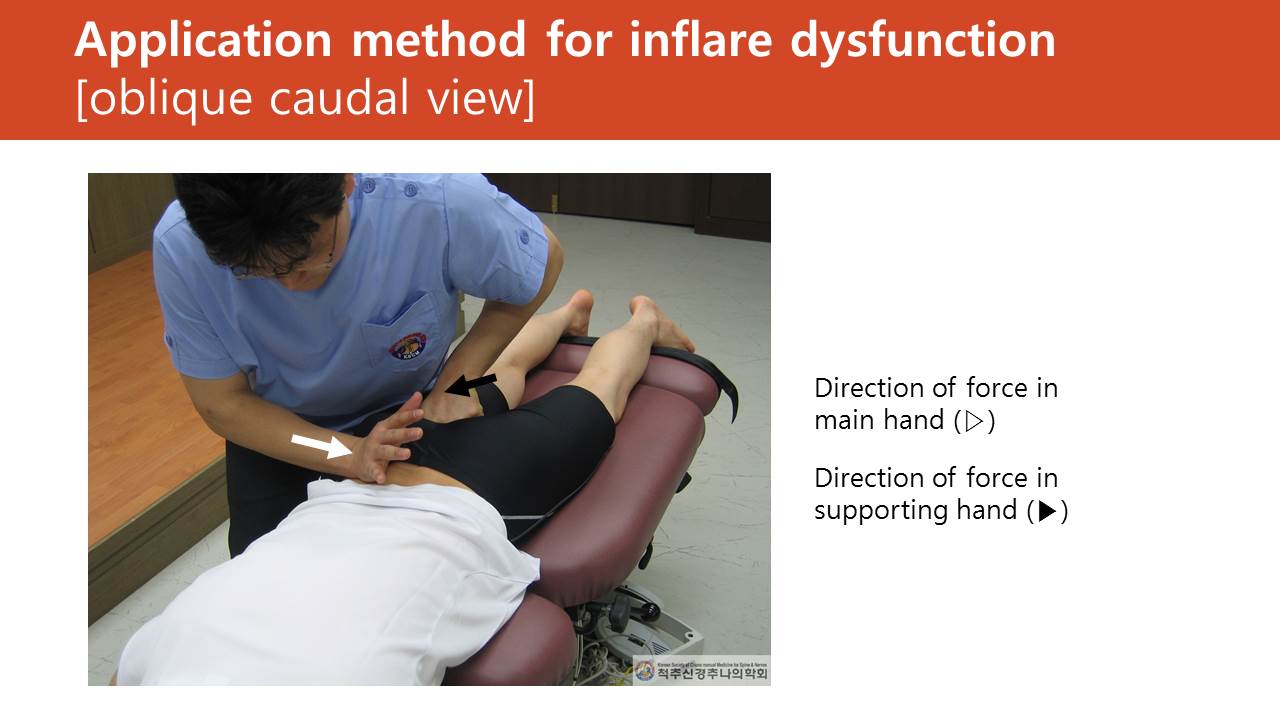

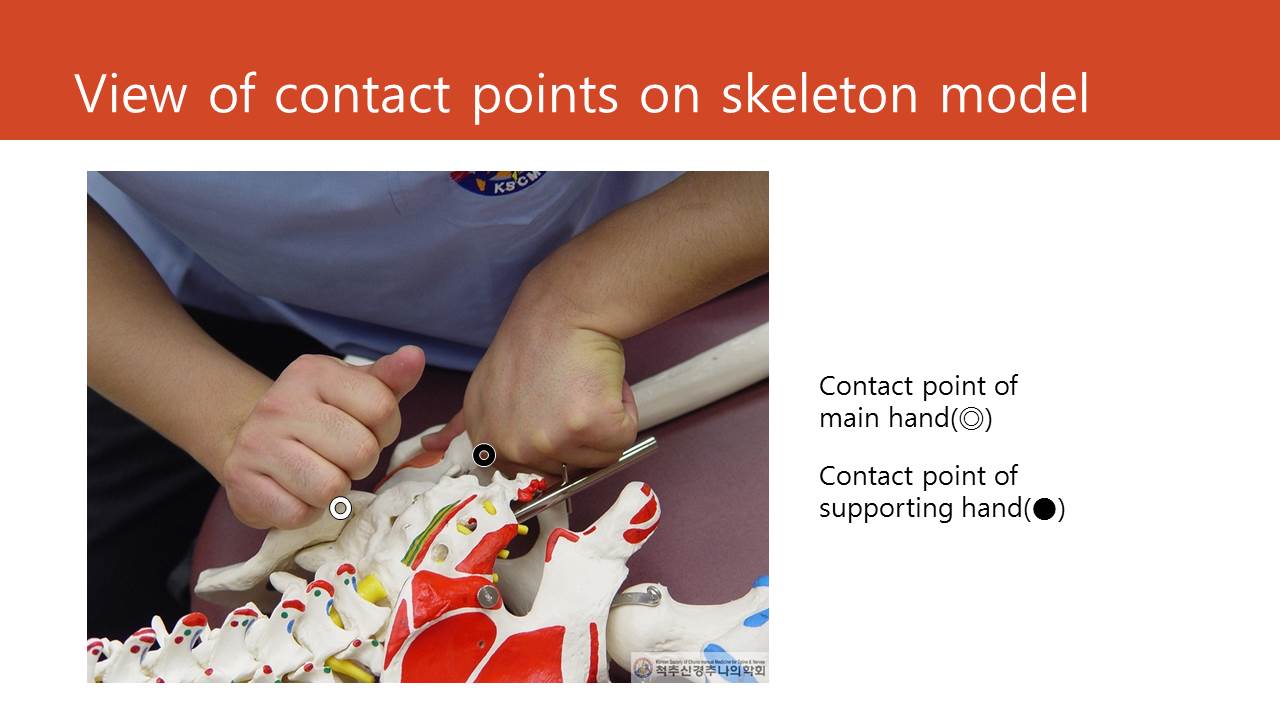


**11. [Mandatory technique] Prone sacral sidebending and rotation dysfunction correction technique [38, 39, 41]**

1) Overview

| Indications | Lt. sidebending with Rt. rotation / Rt. sidebending with Lt. rotation of the sacrum (previously referred to as ‘Lt. anterior inferior / Rt. anterior inferior dysfunction’) |
| --- | --- |
| Patient position | Lies prone |
| Physician position | Stands in fencing stance on the ipsilateral side |
| Main hand | Contacts the medial side of the posterior superior iliac spine (PSIS) contralateral to the sacrum inferior dysfunction with the pisiform bone area of the cephalad hand |
| Supporting hand | Contacts the sacral notch contralateral to the sacrum inferior dysfunction with the pisiform bone area of the caudal hand |

2) Application method


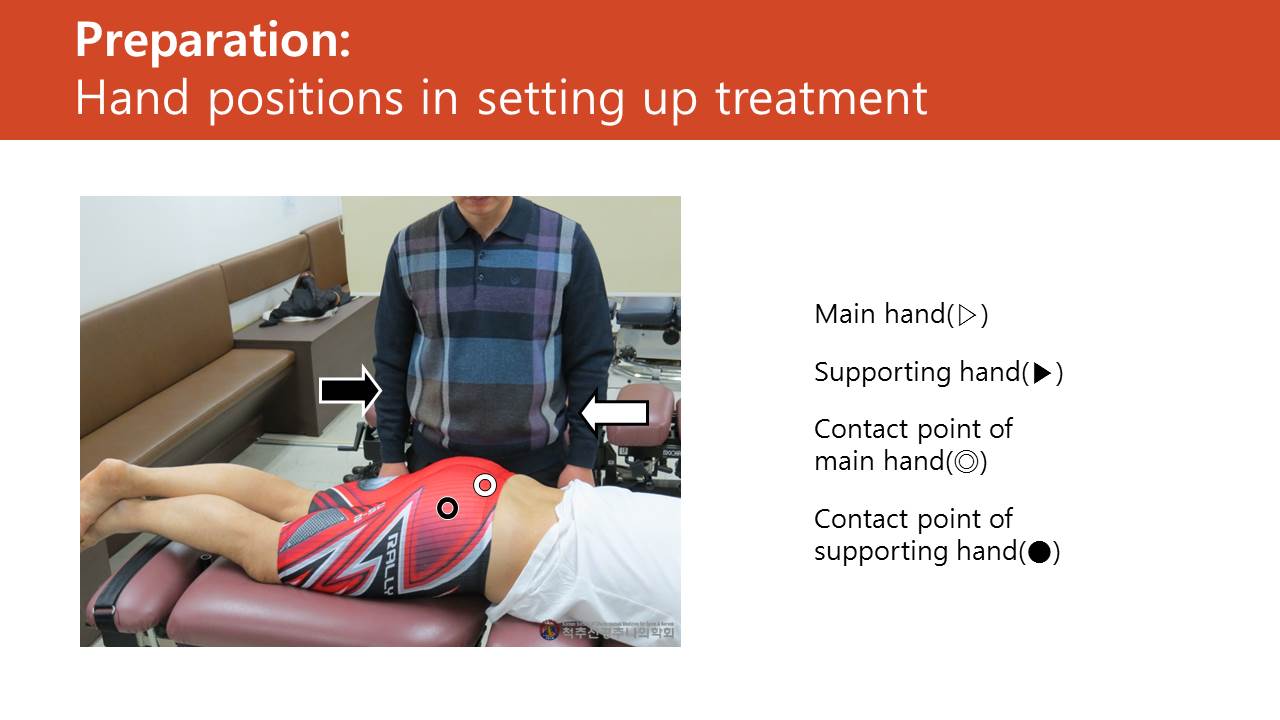

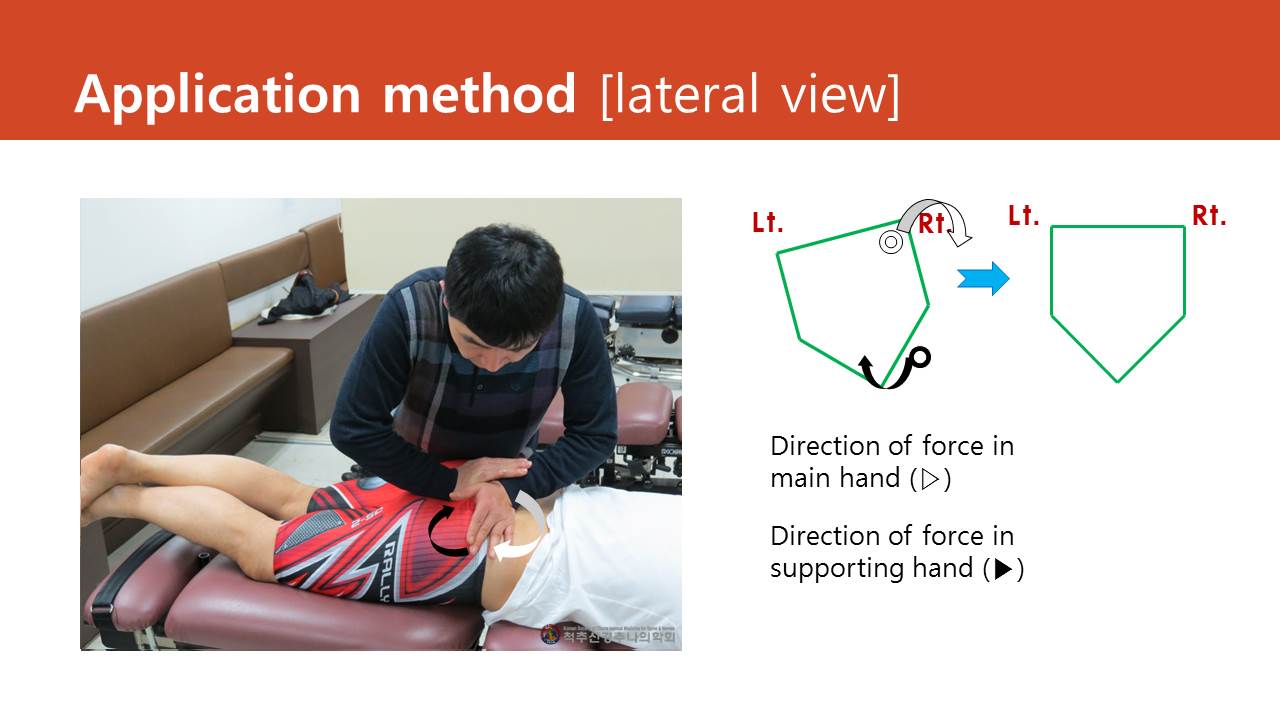

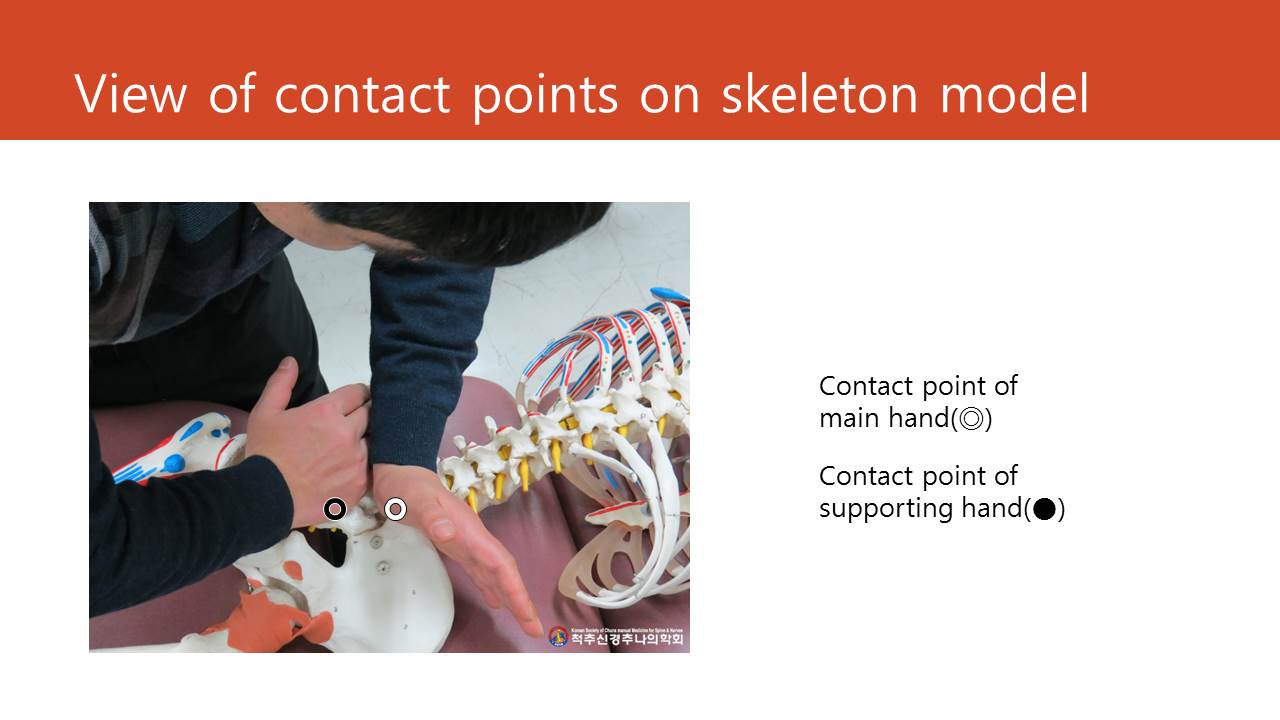


**12. [Selective technique] Prone sacral dysfunction correction technique (extension or flexion) [38, 39, 41]**

1) Overview (flexion dysfunction)

| Indication | Flexion dysfunction of the sacrum (previously referred to as ‘posterior dysfunction of the apex of the sacrum’) |
| --- | --- |
| Patient position | Lies prone |
| Physician position | Stands in fencing stance facing the caudal direction |
| Main hand | Contacts the inferior portion of the sacrum with the heel of the medial hand |
| Supporting hand | Supports the main hand by covering the fingers with the lateral hand |

2) Overview (extension dysfunction)

| Indication | Extension dysfunction of the sacrum (previously referred to as ‘posterior dysfunction of the base of the sacrum’) |
| --- | --- |
| Patient position | Lies prone |
| Physician position | Stands in fencing stance facing the cephalad direction |
| Main hand | Contacts the sacrum base with the thumb of the medial hand |
| Supporting hand | Supports the main hand by covering the thumb with the lateral hand |

3) Application method


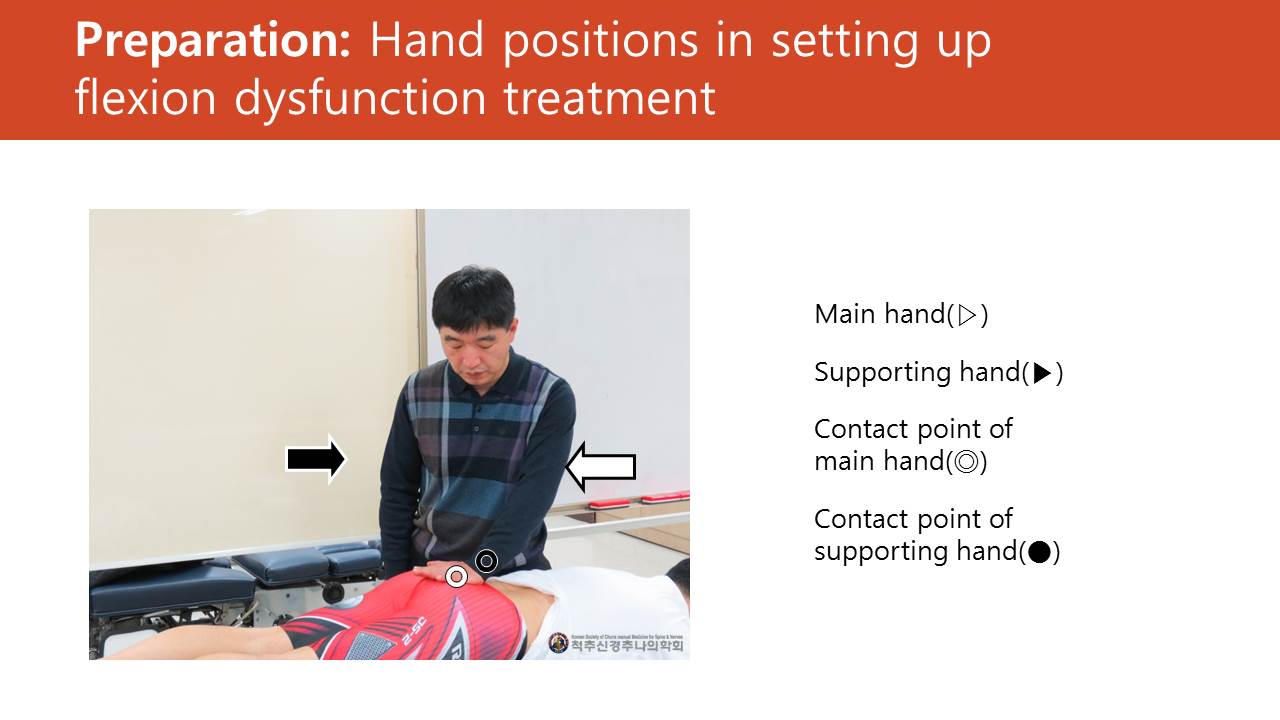

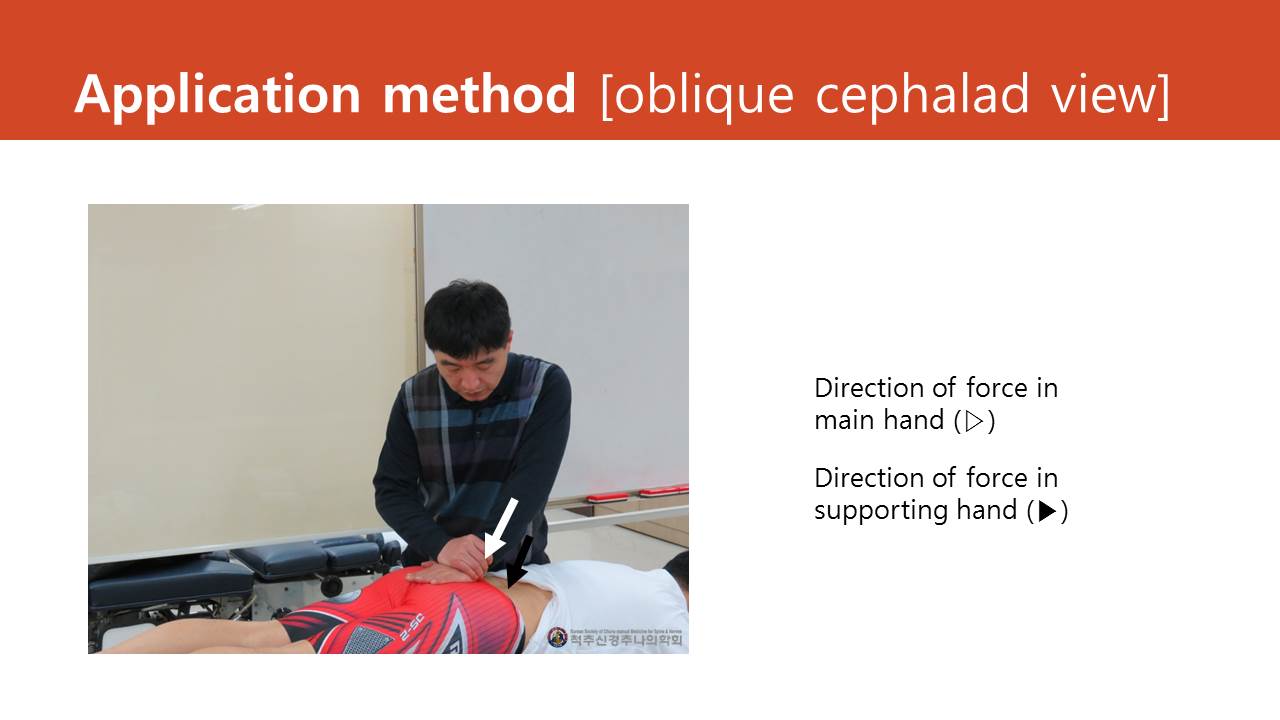

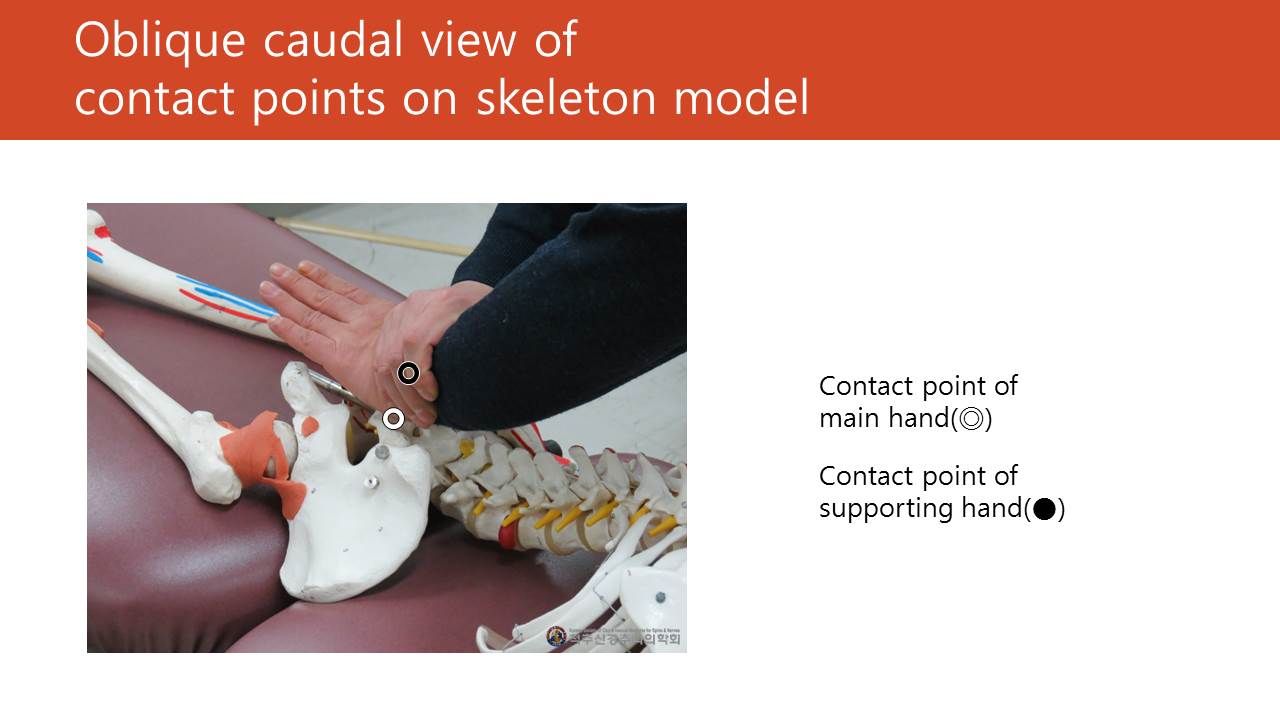

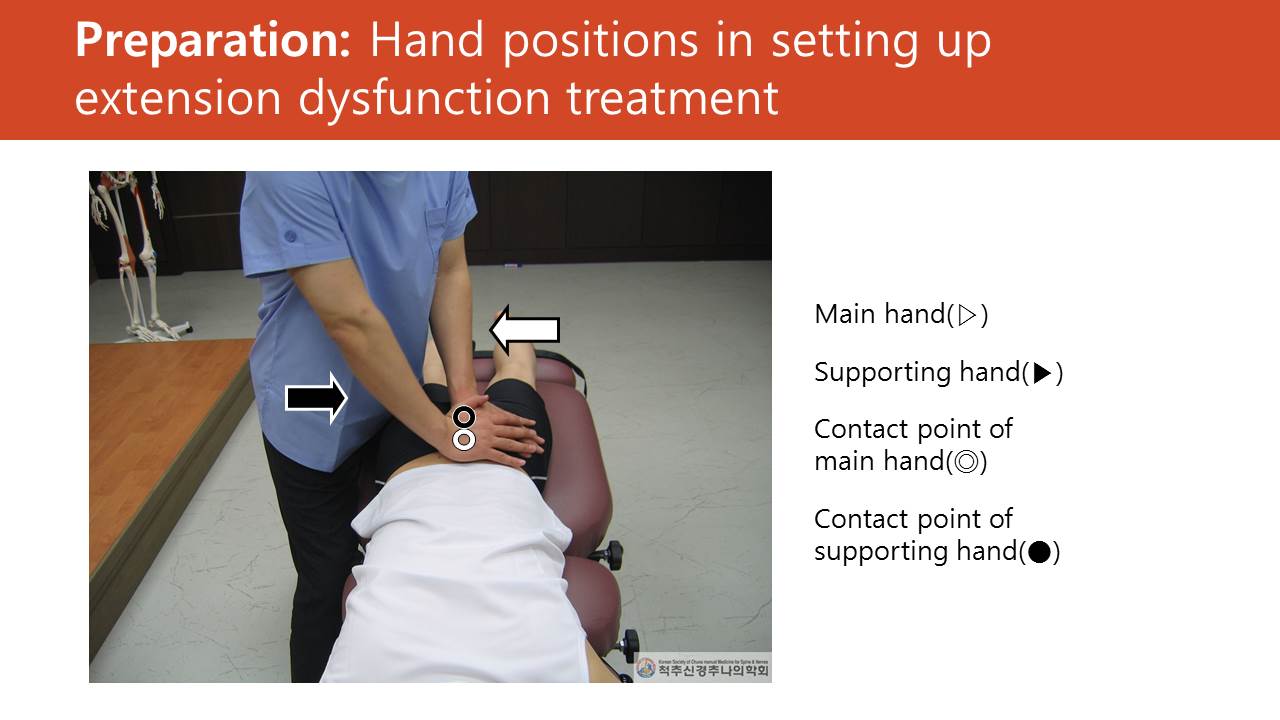

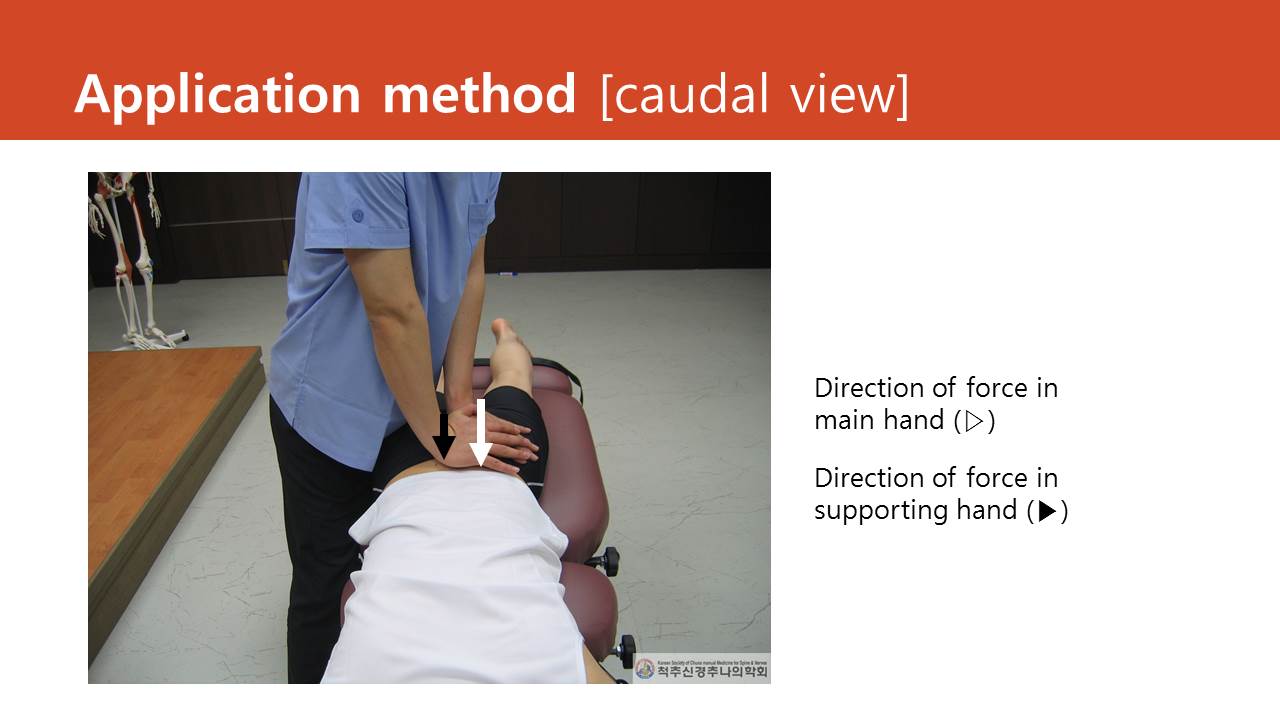

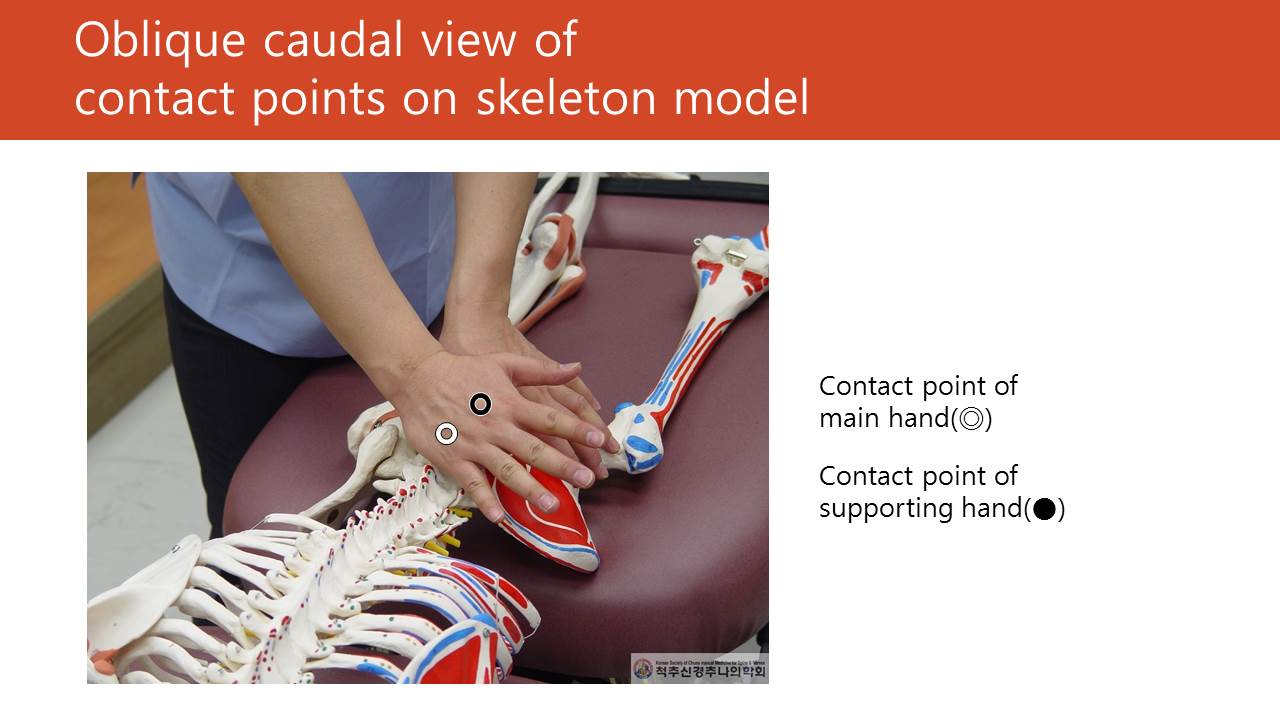


**Reference**

38. Korean Society of Chuna Manual Medicine for Spine & Nerves: *Chuna Medicine:* Seoul: Korean Society of Chuna Manual Medicine for Spine & Nerves; 2014.

39. The Society of Korean Rehabilitation: *Oriental Rehabiliation Medicine:* 3rd ed. Seoul: Koonja Publisher; 2011.

40. DeStefano LA: *Greenman's Principles of Manual Medicine:* 5th ed. Philadelphia, PA: Lippincott Williams & Wilkins; 2016.

41. Bergmann TF, Peterson DH: *Chiropractic Technique: Principles and Procedures:* 3rd ed. St. Louis, MO: Mosby, Inc; 2010.
